# Genome Dynamics of Hybrid *Saccharomyces cerevisiae* During Vegetative and Meiotic Divisions

**DOI:** 10.1534/g3.117.1135

**Published:** 2017-09-15

**Authors:** Abhishek Dutta, Gen Lin, Ajith V. Pankajam, Parijat Chakraborty, Nahush Bhat, Lars M. Steinmetz, Koodali T. Nishant

**Affiliations:** * School of Biology, Indian Institute of Science Education and Research Thiruvananthapuram, Trivandrum 695016, India; † Genome Biology Unit, European Molecular Biology Laboratory, 69117 Heidelberg, Germany; ‡ Department of Genetics, Stanford University, California 94305; § Stanford Genome Technology Center, Palo Alto, California 94304; ** Center for Computation, Modelling, and Simulation, Indian Institute of Science Education and Research Thiruvananthapuram, Trivandrum 695016, India

**Keywords:** mutation rate, hybrid yeast, loss of heterozygosity, meiosis, mitotic recombination

## Abstract

Mutation and recombination are the major sources of genetic diversity in all organisms. In the baker’s yeast, all mutation rate estimates are in homozygous background. We determined the extent of genetic change through mutation and loss of heterozygosity (LOH) in a heterozygous *Saccharomyces cerevisiae* genome during successive vegetative and meiotic divisions. We measured genome-wide LOH and base mutation rates during vegetative and meiotic divisions in a hybrid (S288c/YJM789) *S. cerevisiae* strain. The S288c/YJM789 hybrid showed nearly complete reduction in heterozygosity within 31 generations of meioses and improved spore viability. LOH in the meiotic lines was driven primarily by the mating of spores within the tetrad. The S288c/YJM789 hybrid lines propagated vegetatively for the same duration as the meiotic lines, showed variable LOH (from 2 to 3% and up to 35%). Two of the vegetative lines with extensive LOH showed frequent and large internal LOH tracts that suggest a high frequency of recombination repair. These results suggest significant LOH can occur in the S288c/YJM789 hybrid during vegetative propagation presumably due to return to growth events. The average base substitution rates for the vegetative lines (1.82 × 10^−10^ per base per division) and the meiotic lines (1.22 × 10^−10^ per base per division) are the first genome-wide mutation rate estimates for a hybrid yeast. This study therefore provides a novel context for the analysis of mutation rates (especially in the context of detecting LOH during vegetative divisions), compared to previous mutation accumulation studies in yeast that used homozygous backgrounds.

Most diploid organisms in nature possess heterozygous genomes. In budding yeast, homozygosity is thought to be the default state as seen from wild isolates that come from environments that are undisturbed by humans (Magwene 2014). Isolates of *Saccharomyces cerevisiae* from human-associated (industrial, agricultural, or clinical) environments are observed to have low (<1000 SNPs) to high (>30,000 SNPs) levels of heterozygosity (Argueso *et al.* 2009; Borneman *et al.* 2011; Magwene *et al.* 2011; Magwene 2014). These heterozygosities may be generated due to the accumulation of heterozygous mutations during vegetative divisions, by outcrossing during infrequent sexual cycles [once every 50,000–100,000 mitotic generations (Ruderfer *et al.* 2006), but see Kelly *et al.* 2012] or hybridization events mediated through human activities. The heterozygosity may be further maintained in natural environments by selection. On the other hand, mitotic recombination during vegetative divisions, intratetrad mating, which is common during *S. cerevisiae* meiosis, or selection on genetic variants may contribute to the loss of heterozygosity (LOH).

The baker’s yeast *S. cerevisiae* can undergo vegetative and meiotic divisions rapidly under laboratory conditions. The short generation time and small genome size of *S. cerevisiae* (12 Mb) facilitate analysis of genotypic changes through mutation and recombination processes over a large number of generations (Lynch *et al.* 2008; Halligan and Keightley 2009; Nishant *et al.* 2009). Most mutation accumulation studies in yeast and other microbes involve propagation of isogenic asexual lineages (Lynch *et al.* 2008; Lee *et al.* 2012), though sometimes the sexual cycle has also been incorporated (Nishant *et al.* 2010). The effect of natural selection is minimized through the use of inbred lines and bottlenecks at each generation where a limited number of individuals are randomly selected to produce the next generation. However, the homozygosity of such isogenic lines can mask the detection of genotypic changes induced by mitotic crossovers, gene conversions, and other types of DNA repair processes. Intra and interspecific hybrids of yeast and other organisms have been analyzed during experimental evolution. In addition, a recent study analyzed mutation accumulation during asexual propagation in the microcrustacean *Daphnia* that is naturally heterozygous (Flynn *et al.* 2017). Such hybrid genomes when propagated show large-scale aneuploidies, gross chromosomal rearrangements, and LOH that create a more homogeneous genome (Antunovics *et al.* 2005; Querol and Bond 2009; Burke *et al.* 2010; Morales and Dujon 2012; Dunn *et al.* 2013; Stelkens *et al.* 2014; Flynn *et al.* 2017). Much of this drive toward homogeneity is due to the selection on heterozygous alleles and to purge out genetic incompatibilities (Greig *et al.* 2002; Dunn *et al.* 2013; Wolfe 2015). Similar changes in copy number and genome rearrangements mediated by mitotic recombination have been observed during experimental evolution of isogenic *S. cerevisiae* (Hansche *et al.* 1978; Dunham *et al.* 2002). The mechanisms and distributions of mitotic recombination events are well characterized in yeast (Lee *et al.* 2009; Rosen *et al.* 2013; St Charles and Petes 2013; Yin and Petes 2013; Yim *et al.* 2014). But it is not clear to what extent genotypic changes occur in hybrid yeast over a large number of generations through these somatic DSB repair processes especially when selection is minimized. There are also no measures of mutation rates associated with mitotic and meiotic divisions in hybrid yeast.

We experimentally measured genome-wide LOH and base mutations in an artificial *S. cerevisiae* hybrid strain (S288c × YJM789) propagated through successive vegetative and meiotic divisions. External selection was minimal with the only constraint that the meiotic lines sporulate efficiently and that the spores are viable. The S288c/YJM789 hybrid has ∼60,000 heterozygous SNP markers distributed uniformly across the genome (Wei *et al.* 2007; Mancera *et al.* 2008). Since these SNPs are well characterized, we used them to track genotype changes occurring in the S288c/YJM789 hybrid during vegetative and meiotic divisions. We addressed the following questions: (1) What is the extent and pattern of LOH during vegetative and meiotic divisions? (2) Is heterozygosity preserved on specific chromosomal regions and are there potential fixation biases toward the parental S288c or YJM789 alleles during LOH? (3) How does heterozygosity affect phenotypes such as spore viability? (4) What is the mutation rate associated with vegetative and meiotic divisions in the S288c/YJM789 hybrid?

We observed that LOH through intratetrad mating was rapid in the meiotic lines (70% loss in three generations of meiosis) and associated with improved spore viability. A few of the vegetative lines showed extensive LOH suggesting the occurrence of abortive meiosis and return to growth events during vegetative propagation in laboratory conditions. The base mutation rates in the *S. cerevisiae* S288c/YJM789 hybrid were similar to previous estimates in other *S. cerevisiae* strains suggesting the S288c/YJM789 hybrid is not mutagenic.

## Materials and Methods

### Media and strains

The meiotic (M) and vegetative (V) lines were grown on either YPD (yeast extract, peptone, and dextrose) or synthetic complete (SC) media at 30° (Rose *et al.* 1990). For inducing meiosis, diploids cells were patched on sporulation media (Argueso *et al.* 2004). After 72 hr on sporulation media, tetrads were isolated on YPD or SC using a Zeiss dissection microscope. To generate the parent diploid, cells from overnight patches of YJM789 (*MATα*, *ho*::*hisG lys2*, *cyh*) and S288c (*MATa*, *ho*, *lys5*) were crossed on SC plates to form diploids. They were then streaked on YPD and 12 single colonies were picked and patched on sporulation plates. After 3 d on sporulation media, one of the single colonies that sporulated was stocked and labeled as the parent diploid hybrid strain (KTY162). The KTY162 strain was used to generate the V and M lines.

### DNA extraction and sequencing

Diploid colonies or spore colonies from tetrads were independently cultured overnight at 30° in YPD liquid medium. Genomic DNA was extracted from each culture using the PrepEase DNA isolation kit from Affymetrix following the manufacturer’s protocol. Whole genome sequencing was performed on Illumina HiSequation 2500 machines at Fasteris SA, Switzerland.

### Read mapping, genotyping of whole-genome sequencing data

The sequence reads were mapped to the S288c genome (version 64-1-1, 2011) using bowtie2 (version 2.1.0) (Langmead and Salzberg 2012). Uniquely mapped reads were only considered for the SNP calling (duplicate reads were removed using picardtools). SNPs defined in Mancera *et al.* (2008) were used for all analysis. In order to reduce misalignment due to indels, we performed a local indel realignment after mapping the reads to the reference genome using GATK IndelRealigner. SNPs were called with multiple samples for M line and V lines using GATK unified genotype caller. R package was used for data visualization and downstream statistical analysis. To detect conserved fixed SNPs in the M lines with high spore viability, we subsetted the genotype matrix of all lines from M5 onwards and collapsed consecutive markers that have no change in genotype across all samples. Markers with same genotype across all samples were identified as the boundaries of conserved regions.

### Analysis of LOH tracts

LOH tracts from sequencing of V1_57, V3_57, V4_57, V5_57 diploids were compared with the sequence data from the four haploid spores from these lines. None of the diploid LOH tracts supported by 10 or more SNPs were invalidated when compared with the haploid sequence data from the V1_57, V3_57, V4_57, V5_57 lines. But for LOH tracts supported by <10 SNPs (and especially those supported by only two or three SNPs), some of the SNPs were sometimes called differently in the diploid and haploid data sets (Supplemental Material, Figure S1). This problem may be due to genotyping issues in the diploid sequence data. Therefore, LOH tracts were called in all the V_57 diploid lines only if supported by 10 or more SNPs.

### Analysis of new mutations

To detect new mutations from the M and V lines sequence data sets, we sequenced the parent diploid as well as the S288c and YJM789 strains used to generate the parent diploid. We recalibrated the base qualities in these bam files (generated using alignment with the S288c reference genome) using GATK. SNP positions where the coverage deviates from the median coverage of the sample (due to copy number variation or mapping issues) were excluded. More specifically, at a position, if coverage/median coverage >1.65 || <0.35, the SNP is filtered away. We also filtered away SNPs where QD (Quality by depth) ≤10. For each sample at each potential SNP, we consider the SNP as a mutation if it differs from the genotype in the parent diploid. We checked that it follows a non-mendelian inheritance pattern from the parents (YJM789 and S288c), and this corrects for potential genotyping error in the parent diploid. Next we filtered away sites if the genotype call is not optimal (Genotype quality <30) in either the sample itself, or any of the parent diploid, S288c, or YJM789 strains. All new mutations were verified by Sanger sequencing.

### Data availability

All vegetative and meiotic lines listed in Table S1 are available upon request. Sequence data are available from National Center for Biotechnology Information Sequence Read Archive under accession number: SRP098673. The Data S1, SNP segregation files, and the custom R scripts are available online at the Dryad digital repository (http://dx.doi.org/10.5061/dryad.s14m0).

## Results

### Whole genome sequencing analysis of the S288c/YJM789 hybrid vegetative and meiotic lines

**Figure 1:**
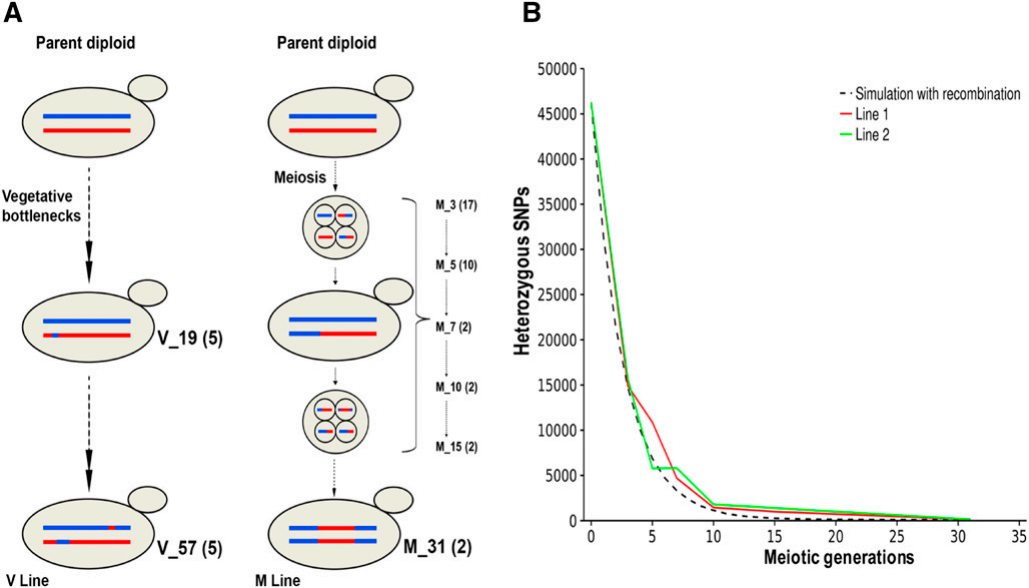
(A) Experimental set up of the vegetative (V) and meiotic (M) lines from the parent diploid. The numbers in brackets indicate the number of V and M lines available at the vegetative bottleneck (V_19, V_57) or meiotic generations (M_3, M_5, M_7, M_10, M_15, M_31). LOH events are observed in both V and M lines. (B) LOH in the M lines following each generation of meiosis and intratetrad mating. Continuous lines (red and green) show the observed number of heterozygous SNPs in two M lines (Line 1 and 2). Black dashes show the number of heterozygous SNPs expected assuming wild-type *S. cerevisiae* recombination rates.

Vegetative and meiotic lines of the S288c/YJM789 parent diploid strain were set up as described in *Materials and Methods* (Figure 1A and Nishant *et al.* 2010). For the meiotic lines, 20 diploid colonies derived from the parent diploid were patched on sporulation media for 3 d and a single complete tetrad from each colony was isolated. Each tetrad was placed on rich media and the germinated spores of opposite mating type mated to form diploids. The resulting colony was then sporulated and the bottleneck repeated. These lines were labeled M1_N to M20_N where N indicates the number of meioses. Most of the M lines showed reduced sporulation after successive rounds of meiosis and could not be propagated with a 3 d sporulation schedule. After seven generations of meiosis and intervening 140 vegetative divisions, only two lines could be maintained as the rest could not sporulate in 3 d (Figure 1A). The two lines were further continued until M_31 (corresponding to 31 generations of meiosis). Whole genome sequence data were obtained from the two lines after the third (M1_3, M2_3), fifth (M1_5, M2_5), seventh (M1_7, M2_7), 10th (M1_10, M2_10), 15th (M1_15, M2_15), and 31st (M1_31, M2_31) generation of meiosis. Six additional lines after five generations of meiosis (M3_5, M5_5, M6_5, M7_5, M8_5, M9_5) were also sequenced to increase the sample size. To accurately genotype heterozygous sites, sequencing was performed on the diploid colonies as well as on the haploid spores obtained by sporulating the diploids (File S1). In parallel, 12 vegetative (V) lines were bottlenecked to single cells from a colony, every 2 d (20 generations). These were initially propagated for a total of 19 bottlenecks (380 generations) that correspond to the amount of time taken for the M_7 lines. These lines were labeled V1_N to V12_N, where N indicates the number of bottlenecks. Five of these lines were propagated further for a total of 57 bottlenecks (1140 mitotic generations) that correlate with the same length of time as the M_31 lines. But the number of mitotic generations in the M_31 lines (620) is fewer due to the intervening meiotic divisions. The V_57 lines (V1_57, V3_57, V4_57, and V5_57) were also sequenced as diploids as well as haploid spores. Due to a recessive lethal mutation in V2_57 (49% spore viability, Table 1), sequencing of haploid spores was not performed for this line. Sequencing details for all M and V lines are in Table S1. The average sequencing depth was ∼70× (Table S1).

**Table 1:** Heterozygous SNP counts from sequencing of diploid M and V lines

Line	Het SNPs	N	S.V (%)	*P* value
M line common SNPs	46,281			
M1_3	14,890	120	92	0.0021
M1_5	10,878	117	94	<0.0001
M1_7	4,688	120	96	<0.0001
M1_10	1,453	120	97	<0.0001
M1_15	995	120	94	<0.0001
M1_31	146	120	93	<0.0001
M2_3	15,655	117	94	<0.0001
M2_5	5,785	119	92	0.0007
M2_7	5,800	119	95	<0.0001
M2_10	1,802	120	98	<0.0001
M2_15	1,401	120	100	<0.0001
M2_31	135	120	94	<0.0001
M3_5	8,912	100	92	0.0005
M5_5	12,345	120	93	<0.0001
M6_5	12,383	120	92	0.0005
M7_5	14,993	120	95	<0.0001
M8_5	10,736	120	93	<0.0001
M9_5	12,825	120	92	0.001
V line common SNPs	47,954			
V1_57	47,026	120	87	0.4
V2_57	46,573	120	49	<0.0001
V3_57	31,580	120	87	0.55
V4_57	44,101	120	90	0.02
V5_57	37,276	120	90	0.026

S.V (%) indicates percentage spore viability for M and V lines. Statistical significance of differences in spore viability between parent diploid (85% spore viability from 180 tetrads) and the M and V lines were determined using the *P* values from Fisher’s test. N: number of tetrads analyzed for spore viability.

### Meiotic lines show almost complete LOH and improved viability

Intratetrad mating of spores is expected to reduce heterozygosity in the M lines (File S2; Knop 2006; Nishant *et al.* 2010). Sequence information from diploid colonies of the two M lines, line 1 (M1_3, M1_5, M1_7, M1_10, M1_15, M1_31) and line 2 (M2_3, M2_5, M2_7, M2_10, M2_15, M2_31), were analyzed for heterozygous SNP markers (Table S2). We focused on 46,281 SNPs that could be called out from all the M lines.

**Figure 2:**
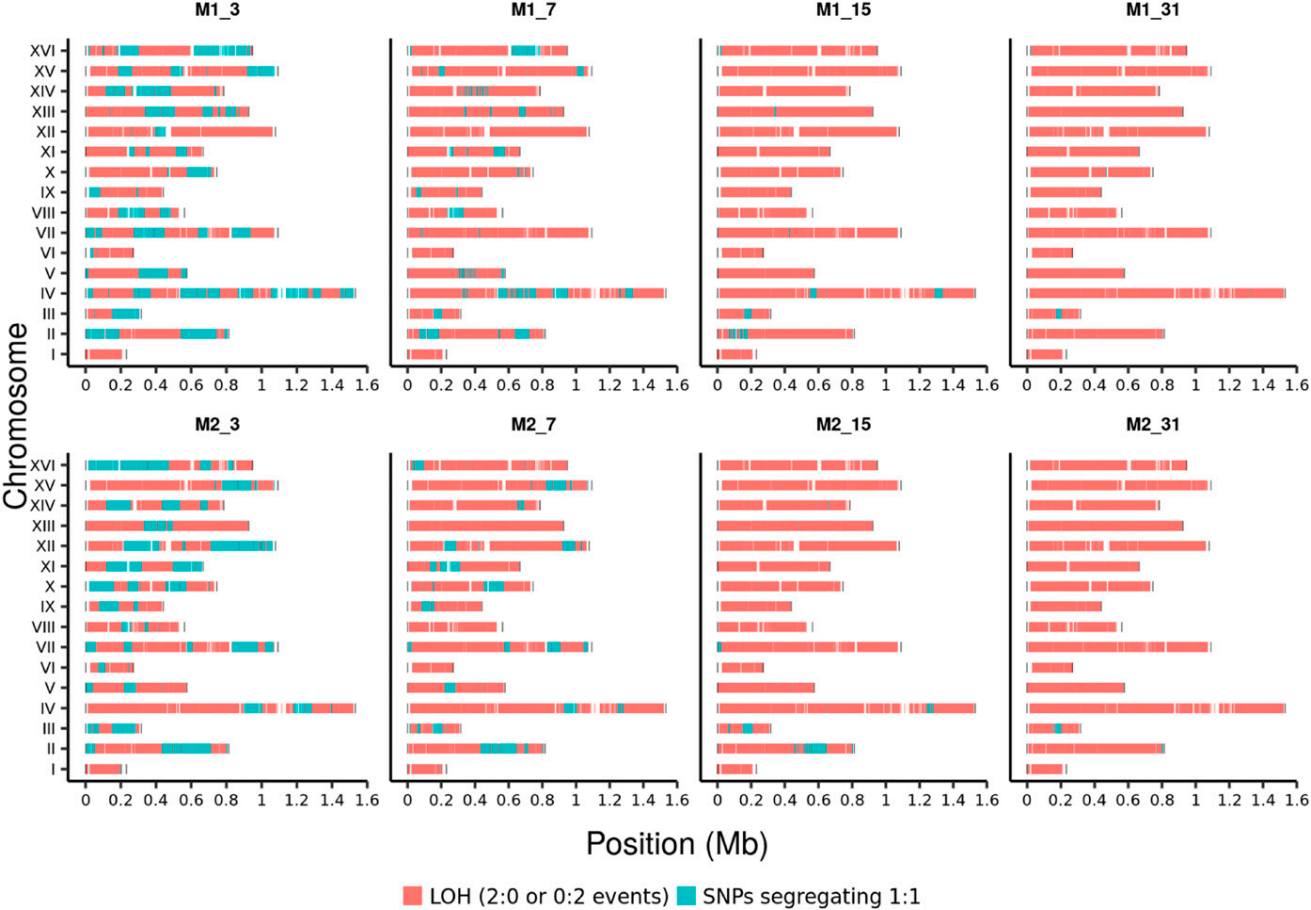
Genome-wide distribution of SNPs in the two M lines (1 and 2) after 3rd, 7th, 15th, and 31st generation of meiosis. Homozygous SNPs are shown in orange while heterozygous SNPs are shown in cyan. More than 99% of the heterozygous sites are fixed by M_31.

The number of heterozygous SNPs rapidly declined with increasing number of meiotic generations (Figure 1B and Table 1). We traced the lineage of each of the 46,281 SNPs for two M lines (1 and 2). By the third round of meiosis, ∼30% of the SNPs were heterozygous and by the seventh round it was ∼10% in these two lines (Figure 2 and Table 1). M1_7 and M2_7 contained 4688 and 5800 heterozygous SNPs, respectively.

We were curious to know whether all heterozygous segregating sites would eventually be lost in the M lines (except for the *MAT* locus, which is under selection). To address this question, we propagated the two lines until M_31. Sequence analysis of M1_31 and M2_31 showed the presence of 146 and 135 heterozygous sites, respectively (Table 1 and Table S2). Most of the heterozygous SNPs in M1_31 (85 out of 146) and M2_31 (102 out of 135) were in proximity to the *MAT* locus on chromosome III, suggesting that they were retained in the heterozygous state because of passive linkage to the *MAT* locus. The rate of LOH was similar for the two lines and maximal in the initial rounds of meiosis when heterozygous markers were maximum. The rate of LOH gradually declined in subsequent generations of meiosis and intratetrad mating. Similar results were observed from the sequencing data of haploid spores from the M lines (File S1 and Table S3).

Since meiotic recombination parameters of the S288c/YJM789 hybrid are well characterized, we mathematically analyzed the LOH in the two M lines (1 and 2) by incorporating high-resolution genome-wide meiotic crossover data of the S288c/YJM789 hybrid (File S2). The M lines are expected to retain on average 98 SNPs by the 31st meiotic generation. This number is close to the experimentally observed number of heterozygous sites in the two M lines. The excess of heterozygous alleles in the M lines compared to the expectation may be because of altered recombination (enhanced) beyond M_5 as the lines become extremely homozygous or due to factors other than recombination (*e.g.*, chromosomal structural features or genomic loci besides the *MAT* locus that affect mating) that contribute to the maintenance of residual heterozygosity in the M lines. Besides the *MAT* locus, the distribution of the residual heterozygous sites between the two lines were unique (Figure 2).

The M lines showed rapid fixation of alleles toward the S288c or YJM789 state. In order to statistically test biased fixation toward S288c or YJM789 alleles, we used the sequence data from the eight M5 lines (M1_5, M2_5, M3_5, M5_5, M6_5, M7_5, M8_5, M9_5). Five of the eight M_5 lines showed excess fixation toward the YJM789 alleles. Across these eight lines, an average of 18,276 SNPs were fixed toward YJM789 and 16,897 SNPs were fixed toward S288c (Figure S2A and Table S2). We simulated five successive rounds of meiosis with intraspore mating, using crossover locations from Mancera *et al.* (2008) and Liu *et al.* (2014) to test if the fixations toward S288c and YJM789 SNPs are significant. We tabulated the number of fixed YJM789 SNPs in the eight M_5 lines, over 5000 simulations. The average number of YJM789 fixed SNPs in the eight M_5 lines (18,276 SNPs) was within the expected range based on simulation data and suggest these biased fixations are not statistically significant (Figure S2B). Asymmetric fixation of alleles derived from either parent in a hybrid has been observed previously (Tang *et al.* 2010).

The S288c × YJM789 hybrid has a spore viability of 85% unlike the S288c diploid that has a spore viability >97% (Table 1; McCusker *et al.* 1994). The presence of ∼60,000 SNPs along with indels can create incompatibilities in the S288c/YJM789 hybrid causing reduced spore viability (Wei *et al.* 2007). Meiotic lines at M_3 and beyond showed significantly improved spore viability (>92%) compared to the parent diploid (Table 1). This result is interesting because it suggests that the significant loss in heterozygosity observed by the third generation of meiosis or fixation of certain S288c and YJM789 alleles may contribute to the improved spore viability in the M lines (see below).

### Variable LOH in hybrid vegetative lines

**Figure 3:**
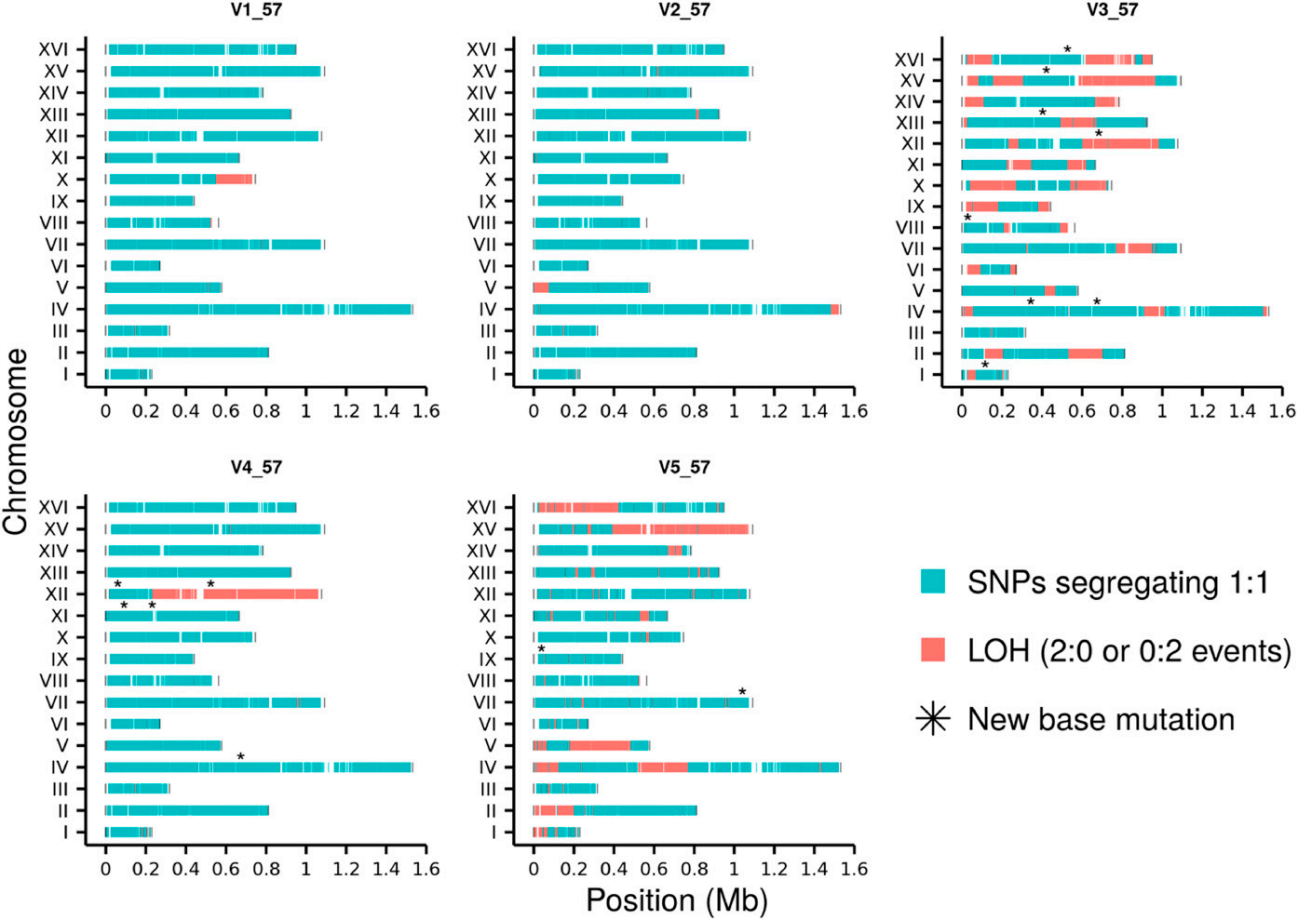
Genome-wide plots of LOH and new base mutations in the five V_57 lines. Regions showing loss of heterozygosity are in orange (2:0 or 0:2). Regions in cyan are heterozygous (1:1). Asterisk (*) symbols show the position of the new base mutations in the V_57 lines.

Hybrid vegetative lines can be used to detect LOH during mitotic divisions. Hybrid *S. cerevisiae* strains have been previously used to map LOH events involving mitotic crossovers and break induced replication (BIR) events on specific chromosomes III, IV, and V and genome wide (Lee *et al.* 2009; Rosen *et al.* 2013; St Charles and Petes 2013; Yin and Petes 2013; Yim *et al.* 2014). These studies have generated considerable insights into the mechanisms and distributions of mitotic recombination. The mitotic recombination events are thought to occur with ∼10^5^-fold less frequency than meiotic recombination and therefore require a selection system for their detection (Lee *et al.* 2009). But it is possible that over a large number of divisions they can cause significant genotypic changes that can be detected and is relevant to measure, given the ratio of mitotic to meiotic cycles in yeast (Ruderfer *et al.* 2006; Kelly *et al.* 2012). Diploid colonies from five vegetative lines (V1–V5) were whole-genome sequenced after 57 bottlenecks (corresponding to the 31st generation of meiosis for the M lines) (Table S4). We focused on the 47,954 SNPs that are common to all the five V lines. In the lines V1_57 and V2_57, only 2–3% of the SNPs showed LOH compared to >99% of the heterozygous sites that became homozygous in the meiotic lines propagated for the same length of time (M_31) (Table 1). In V4_57, 8% of the SNPs showed LOH, which was primarily due to a single large terminal LOH tract on chromosome XII (Figure 3). In the most extreme cases in V3_57 and V5_57, up to 35 and 22% of the heterozygous sites became homozygous. LOH in the V lines likely results from the repair of DNA lesions using the homologous chromosome followed by segregation of sister chromatids. Mitotic crossovers, local gene conversions, BIR, and chromosome loss are implicated in LOH events during mitosis (Paques and Haber 1999; Barbera and Petes 2006).

We analyzed the SNP distribution data in the V_57 lines to map the accumulated LOH tracts genome wide. Sequence data from both the diploid V_57 lines as well as their haploid derivatives (Table S5) were used to call LOH tracts accurately (*Materials and Methods*). Genome-wide distribution of 141 LOH tracts in the V lines is shown in Figure 3 and Figure S3A along with information on chromosomal location and tract sizes (Table S6). Since the LOH tracts in the vegetative lines are summed over 1140 mitotic generations, these tracts may have been generated through multiple DSB repair mechanisms that are difficult to distinguish. The tract sizes showed a broad distribution from <100 bp to over 100 kb (Figure S3, B and C and Table S6). The tracts were on average closer to telomeres than centromeres. The average distance of the LOH tracts to the nearest telomere was 126.2 kb compared to 244.7 kb for the centromere (Table S6). This is consistent with the increase in mitotic recombination rates away from the centromere in *S. cerevisiae* (Mandegar and Otto 2007). Centromere distal regions have also been shown to be more prone to spontaneous LOH events in *S. cerevisiae* (Andersen *et al.* 2008). V1_57, V2_57, and V4_57 showed fewer LOH tracts than V3_57 and V5_57. The long LOH tracts in V1_57, V2_57, and V4_57 lines were primarily terminal as expected to arise during mitotic crossovers or BIR (Figure 3 and Figure S3A). In contrast, V3_57 and V5_57 have frequent long internal LOH tracts that might arise due to high levels of DNA damage and repeated rounds of mitotic recombination events creating chimeric chromosomes that are fixed alternatively toward S288c or YJM789 (Morales and Dujon 2012). The high frequency of long internal LOH tracts also suggest the possibility that V3_57 and V5_57 may have been through a cycle of abortive meiosis and return to growth involving DSB repair (Dayani *et al.* 2011; Laureau *et al.* 2016). Twenty-eight 2:0/0:2 LOH events >100 kb were observed (Table S6) with the largest one ∼827 kb on chromosome XII in V4_57. Chromosome XII has been previously also observed to have large LOH tracts (Magwene *et al.* 2011). The short LOH tracts (<1 kb) are likely to have arisen through local gene conversions not associated with crossovers (Palmer *et al.* 2003; Laureau *et al.* 2016). Chromosomal aneuploidies contributing to LOH were not observed in the V lines (Figure S4), which suggests the S288c/YJM789 hybrid does not show chromosomal instability during mitotic divisions.

During experimental evolution of interspecific hybrids, biased elimination of one of the parent genomes as well as specific genome changes driven by selection are often observed (Antunovics *et al.* 2005; Dunn *et al.* 2013). When the V line’s SNP data were analyzed for biased fixation, 81 LOH tracts were fixed toward S288c compared to 60 tracts toward YJM789 (*P* = 0.092, binomial test) (Table S6). Despite random fixation of the LOH tracts toward S288c or YJM789, we observed more fixed YJM789 SNPs as most of the longer LOH tracts are fixed toward YJM789 alleles (Figure S2A and Table S6).

Unlike the significant improvement in spore viability observed in all meiotic lines, the spore viability of two of the vegetative lines (V1_57 and V3_57) was not significantly different from the parent diploid strain (Table 1). V2_57 showed the presence of a recessive lethal mutation. V4_57 and V5_57 showed improvement in spore viability compared to the parent diploid (0.05 < *P* > 0.01), but the differences were less significant when compared to the meiotic lines (*P* < 0.0001). These results suggest LOH did not affect the spore viability of the hybrid vegetative lines.

### Base mutations in the hybrid vegetative and meiotic lines

While LOH events and intratetrad mating can reduce heterozygosity, new mutations can add to the number of heterozygous sites in the genome. We estimated the number of base substitutions of the S288c/YJM789 hybrid vegetative (V1_57, V2_57, V3_57, V4_57, V5_57) and meiotic lines (M1_31, M2_31) with reference to the parent diploid genome. We detected one mutation in the meiotic line (M1_31) and 15 mutations across three vegetative lines (eight in V3_57, five in V4_57, and two in V5_57) (Table 2). No base substitution mutations were observed in M2_31 and V1_57, V2_57. It is possible that the recessive lethal mutation in V2_57 is caused by an in-del or other structural variation. All the 16 mutations were validated by Sanger sequencing. All the new mutations were heterozygous and did not occur in the coding region of the genome. Only one mutation was shared between two of the vegetative lines V3_57 and V4_57 (C > A on Chr IV). The vegetative lines were propagated for a total of 1140 mitotic divisions (57 bottlenecks) and the 24.04 Mb genome was sequenced at 99.9% sequence coverage. The mutation rates are 2.92 × 10^−10^ for V3_57; 1.82 × 10^−10^ for V4_57, and 0.73 × 10^−10^ for V5_57. The average mutation rate for the three vegetative lines is therefore 1.82 × 10^−10^ per base per division. The average mutation rates in the S288c/YJM789 hybrid are similar to mutation rate estimates from a large set of 145 diploid *S. cerevisiae* vegetative mutation accumulation lines from Zhu *et al.* (2014) (1.67 × 10^−10^ per base per generation). They are also comparable to the homozygous *S. cerevisiae* SK1 diploid mutation rates (2.9 × 10^−10^) in vegetative lines (Nishant *et al.* 2010).

**Table 2:** Genomic locations of the 16 base mutations identified in the M and V lines

Line	Chromosome	Position	Ref Allele	Alt Allele
M1_31	XII	761670	C	G
V3_57	I	115140	A	C
V3_57	IV	342790	C	T
V3_57*^a^*	IV	671895	C	A
V3_57	VIII	29209	T	C
V3_57	XII	681833	T	G
V3_57	XIII	401462	G	A
V3_57	XV	420877	T	C
V3_57	XVI	525859	T	C
V4_57*^a^*	IV	671895	C	A
V4_57	XI	89201	C	A
V4_57	XI	231710	G	T
V4_57	XII	60920	G	T
V4_57	XII	524342	A	C
V5_57	IX	38609	G	C
V5_57	VII	1041202	C	T

a Shared mutation.

To calculate mutation rates for the S288c/YJM789 hybrid meiotic lines, we multiplied the number of mutations observed in the meiotic line by two, as half of the mutations in the meiotic lines are expected to be lost during intratetrad mating (Nishant *et al.* 2010). The base substitution rate for M_31 is estimated to be 1.33 × 10^−10^ per base per division (31 meiotic divisions + intervening 620 mitotic divisions and 99.9% sequence coverage of the 24.04 Mb diploid genome). For comparison, the base substitution rate in *S. cerevisiae* SK1 meiotic lines was 3.9 × 10^−10^ after 50 meiotic divisions and 1000 intervening mitotic divisions (Nishant *et al.* 2010).

Higher mutation rates have been observed in heterozygotes compared to homozygotes based on sequence analysis of parent–progeny in *Arabidopsis* and rice (Yang *et al.* 2015). Since we do not have mutation rate data for *S. cerevisiae* meiotic lines that are homozygous for S288c or YJM789, we cannot infer whether the same is true in *S. cerevisiae* as well. It is also important to note that S288c/YJM789 is an artificial hybrid with uniform distribution of heterozygous markers and these heterozygosities were progressively reduced during the meiotic divisions. In the meiotic lines, heterozygosity is lost by 70% within three generations of meiosis. In addition, the meiotic lines undergo both vegetative and meiotic divisions and there is 50% loss of mutations during intratetrad mating of spores (Nishant *et al.* 2010). It is therefore likely that more mutations were not observed in the S288c/YJM789 hybrid meiotic lines due to the study design (Nishant *et al.* 2010; see *Discussion*). In the vegetative lines, no base mutations were observed in V1_57 and V2_57 that have 2–3% loss of heterozygous SNPs. All of the base mutations were observed in the V3_57, V4_57, and V5_57 lines, which have a significantly higher percentage of LOH events (Figure 3). This is consistent with previous observations in yeast, where the repair of HO endonuclease-induced DSBs causes high mutation frequency proximal to the break site (Strathern *et al.* 1995). But only two of the 16 mutations observed in the vegetative lines were located within the LOH tracts (Figure 3). Since all the 16 mutations are heterozygous, it is possible that the other mutations happened in locations where the mitotic recombination events were repaired using the sister chromatid; the DSB repair through other mechanisms did not leave an LOH signature or the SNP density was not sufficient to detect an LOH. It is also possible that some of the mutations in the LOH tracts may have been fixed in favor of the parent allele, and so the mutation event is not detected. It is also possible that at least for V3_57 and V5_57, mutations may be associated with recombination repair of DSBs during partial entry into meiosis followed by return to growth.

### Heterozygosity determines spore viability in the hybrid meiotic lines

The meiotic lines showed enhanced spore viability. We tested if the improved spore viability is due to the biased fixation toward S288c/YJM789 alleles or due to a general reduction in heterozygosity. We observed specific conserved chromosomal regions that were fixed either toward S288c or YJM789 alleles in the meiotic lines. We identified 20 such regions (Figure S2C). The largest region was observed on chromosome XV, and it showed fixation towards the S288c alleles. We backcrossed M2_15 spores (1104 SNPs and 100% SV) with the parent S288c and YJM789 strains. The backcross increases the heterozygosity and reintroduces the incompatibilities that might have existed in the parent hybrid. The heterozygous SNPs in the M2_15 × S288c cross increased to 22,923 SNPs and in the YJM789 cross to ∼19,838 SNPs. In addition, the backcross with the parent S288c creates an S288c/YJM789 hybrid set for the YJM789 fixed candidate regions, while the backcross with YJM789 created an S288c/YJM789 hybrid set for the S288c fixed candidate regions. In both the crosses, we observed a spore viability of ∼95% (*n* = 60 tetrads), which is significantly higher than that for the wild-type hybrid parent (85%, Fischer exact test, *P* < 0.0001). Similarly, spores from M6_5 (12,383 SNPs, 92% spore viability) crossed with S288c (26,693 SNPs) and YJM789 (17,004 SNPs) showed a similar spore viability of 90%. These results suggest that the regions fixed either in favor of S288c or YJM789 may have no role in the improved spore viability of the M lines. Instead, the spore viability of the M lines is affected by the heterozygosity of the lines. Previous studies have observed a negative correlation of heterozygosity with sporulation efficiency as well as spore viability in *Saccharomyces* wild isolates (Mortimer *et al.* 1994; Liti *et al.* 2006; Cubillos *et al.* 2011; Magwene *et al.* 2011).

**Figure 4:**
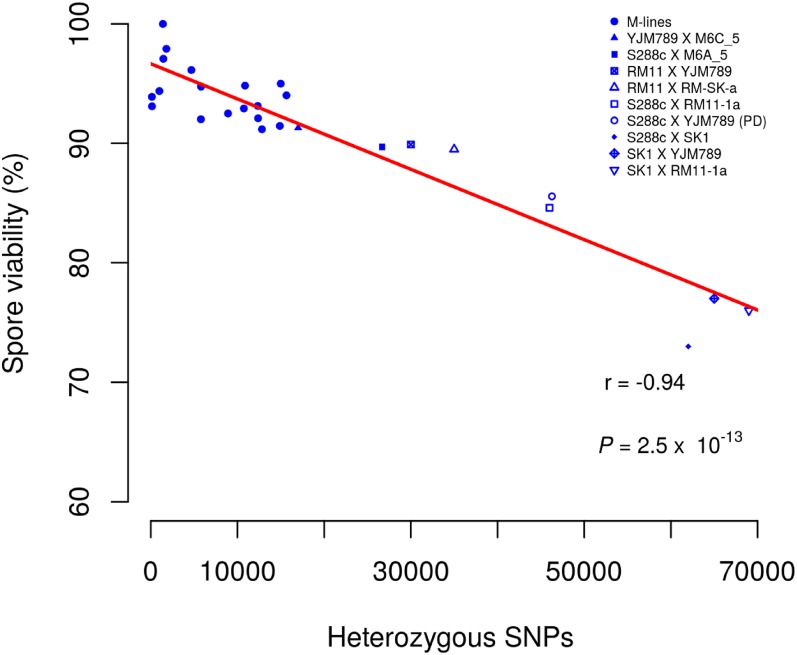
Heterozygosity is negatively correlated with spore viability. The number of heterozygous SNPs and spore viability are plotted against each other for the S288c × YJM789 diploid M lines and for hybrid crosses involving other *S. cerevisiae* strains. Data for the M lines are from Table 1. Data for other hybrid crosses are shown in the text.

We further tested this hypothesis using different *S. cerevisiae* artificial hybrid combinations of S288c, SK1, YJM789, and RM11-1a as well as data from the M lines (Figure 4). The heterozygosity in these hybrids and the M lines is well defined facilitating a calibrated comparison of spore viability with the level of heterozygosity. Among the different *S. cerevisiae* artificial hybrid combinations, the hybrid with the least heterozygosity, *i.e.*, RM11-1a × YJM789 (∼30,000 SNPs; Gresham *et al.* 2006), showed the maximum spore viability (90%). Hybrids with higher heterozygosity, *e.g*., S288c × RM11-1a (∼46,000 SNPs; Qi *et al.* 2009), S288c × SK1 (∼62,000 SNPs; Martini *et al.* 2011), SK1 × YJM789 (∼65,000 SNPs), and SK1 × RM11-a (∼69,000 SNPs), showed lower spore viability (85, 73, 77, and 76% respectively) (Figure 4). Overall a strong negative correlation was observed between heterozygosity and spore viability (*r* = −0.94, *P* = 2.5 × 10^−13^). These observations suggest the high spore viability phenotype of the M lines is due to the reduction in the heterozygous load. Reduced heterozygosites can result in less heteroduplex rejection during recombination repair of DSBs (Chakraborty and Alani 2016). The improved repair outcomes and fewer genetic incompatibilities (*e.g.*, between S288c-Mlh1 and SK1-Pms1; Heck *et al.* 2006) may increase spore viability in homozygous backgrounds.

### Generation of a panel of heterogeneous inbred families (HIFs) from the hybrid meiotic lines

**Figure 5:**
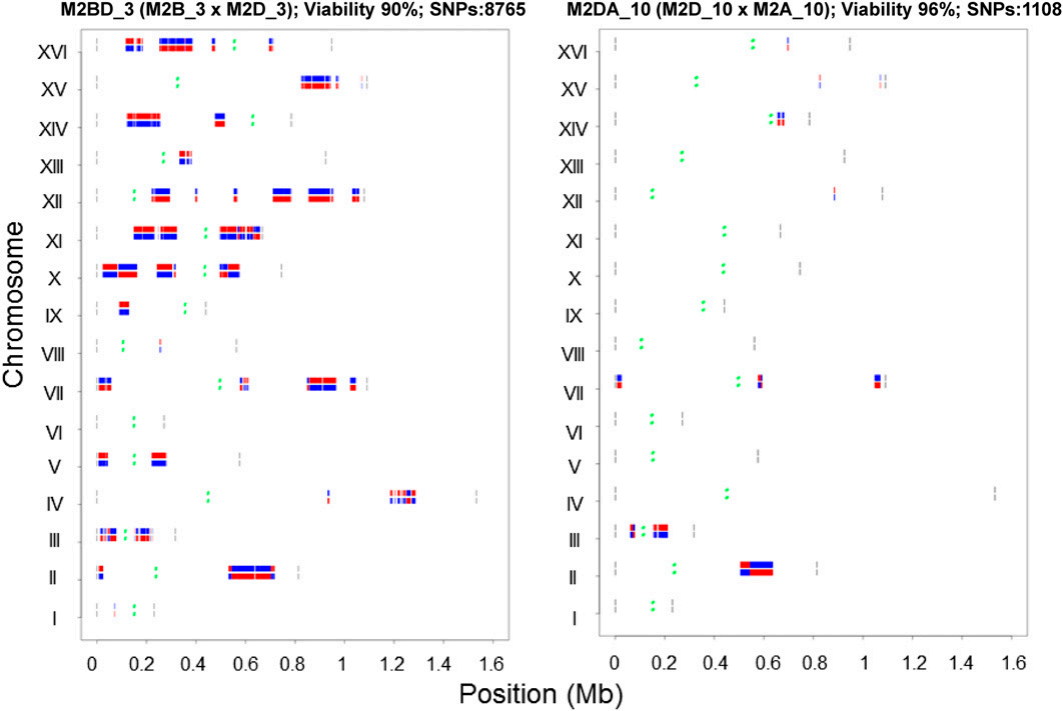
Representative *in silico* genomes generated by crossing spores from M_3 and M_10 lines. White regions on the chromosome are homozygous for S288c or YJM789. Red and Blue indicate S288c and YJM789 SNPs respectively. The diploids generated from the cross have good viability (>90%) and are heterozygous at specific chromosomal regions. The green and gray regions indicate centromere positions and chromosome boundaries, respectively.

Crosses between inbred lines can facilitate fine mapping of QTL (Flint and Mott 2001; Bergelson and Roux 2010). We experimentally determined the mating type of all spores derived from the M lines. These spores were mated *in silico* and the genome sequence information was used to generate a total of 1369 *in silico* diploid genomes (Table S7). These *in silico* diploid genomes show varying numbers of SNP counts and retain heterozygosity at specific regions on the genome [Data S1, available online at the Dryad digital repository (http://dx.doi.org/10.5061/dryad.s14m0)]. The rest of the genome remains homozygous for S288c or YJM789. This collection of 1369 *in silico* diploid genomes represents a panel of HIFs that can be experimentally generated by crossing the haploid spores from the M lines. The distribution of SNPs for two representative HIF strains that were also experimentally analyzed for spore viability is shown in Figure 5. The two diploid strains, M2BD_3 and M2DA_10, were generated by crossing two spores from M2_3 and M2_10 each. They have spore viability ≥90% and different numbers of heterozygous markers. M2BD_3 has 8765 heterozygous SNPs while M2DA_10 has 1108 heterozygous SNPs. Further, M2DA_10 has nine chromosomes (XIII, XI, X, IX, VIII, VI, V, IV, and I) that are completely homozygous while the rest of the chromosomes have heterozygous regions. Such HIF strains can be used to study the effects of varying levels of heterozygosity on different biological processes including spore viability (Figure 5) and meiotic recombination. Since SNP markers are lost as the lines become homozygous, cytological, physical, or biochemical methods can be used to test if genome-wide meiotic recombination is enhanced. The HIF lines are also useful for mapping QTL for traits that differ between S288c and YJM789. The S288c × YJM789 hybrid displays heterosis across many phenotypes including high-temperature growth and sporulation (Steinmetz *et al.* 2002). If a trait of interest segregates in such a diploid, it is relatively easy to identify the causative SNPs as the regions of heterozygosity are limited. If candidate regions for the QTL are already known, one can use the sequence information of the spores from the M lines to generate hybrid diploids that are heterozygous specifically in the candidate regions and thus fine map the QTL (Table S8 in File S1).

## Discussion

An artificial hybrid such as S288c × YJM789 provides a unique opportunity to study the interaction between two independent genomes that have never existed together, in successive vegetative and meiotic generations. Unlike isogenic strains that can be propagated for successive meiotic divisions for large numbers of generations (Nishant *et al.* 2010), we observe that hybrid strains like the S288c/YJM789 pose unique problems during meiotic propagation. Only 2 of the 20 M lines could be propagated beyond the seventh generation of meiosis due to poor sporulation of the other lines. The most likely explanation is that both S288c and YJM145 (the diploid progenitor of YJM789) are poor sporulators (McCusker *et al.* 1994). In comparison, the S288c × YJM789 hybrid efficiently sporulates within 3 d. Reduction in sporulation efficiency of many of the meiotic lines suggest that alleles from both S288c and YJM789 contribute to the good sporulation phenotype of the S288c/YJM789 hybrid. During the meiotic propagation of the lines, some of the alleles contributing to the high sporulation efficiency may be lost through fixation of the opposite allele. Consistent with this, even after five generations of meiosis, the sporulation efficiency of the M_5 lines was variable (Figure S5).

Whole-genome sequence analysis of meiotic lines showed a significant decline in heterozygosity as expected from intratetrad mating. A significant decrease in heterozygosity was also observed in some of the vegetative lines (V3_57, V4_57, V5_57). These results are relevant for interpreting heterozygosity in *S. cerevisiae* populations, since meiosis occurs infrequently in *S. cerevisiae* and most diploids that arise are produced by intratetrad mating (Magwene *et al.* 2011). Previous studies have suggested a very low outcrossing frequency of 0.5 per 10^5^ mitotic divisions (Ruderfer *et al.* 2006). However, a recent study (Kelly *et al.* 2012) shows mating and outcrossing frequency in *S. cerevisiae* is much higher (1 in 100 mitotic divisions). These new estimates of outcrossing can better explain heterozygosity in wild *S. cerevisiae*, given the extent of LOH in a few of the vegetative lines and during intratetrad mating that we observed in this study. Residual heterozygosity observed in these two M lines was conserved only at the *MAT* locus since we were selecting for hybrids that can sporulate. Selection acting on standing genetic variation can cause fixation as well as LOH of linked regions (Burke *et al.* 2010). We show that almost all heterozygous sites in the S288c/YJM789 hybrid yeast genome can be fixed even with minimal external selection. The fixation of SNPs occurred in large blocks after each meiosis consistent with fixation driven primarily by mating comprising recombinant spores (Figure S6, Figure S7, and Figure S8 in File S1).

The fixation of the heterozygous sites in the meiotic lines can occur randomly toward S288c or YJM789 alleles or may show biases, either local or genome wide, especially if selection favoring either of the alleles is involved (*e.g.*, high temperature where the YJM789 genotype grows better; Wei *et al.* 2007). Even without apparent external selection, the presence of two different genomes (S288c and YJM789) in the hybrid could create fixation of either genotype through biased selection. Such regions were observed in the M lines (Figure S2C). The fixation of these specific regions toward S288c and YJM789 alleles may arise due to differences in expression from the S288c and YJM789 alleles of the same gene; epigenetic reasons; preferential initiation of meiotic DSBs from chromosomal regions containing S288c or YJM789 SNPs followed by biased MMR; or selection for good sporulators (Figure S9 in File S2).

Heterozygous SNPs in the S288c/YJM789 hybrid facilitated the analysis of genome dynamics as cells cycle through vegetative divisions. Genetic changes like LOH can uncover mutations in tumor suppressor genes commonly associated with cancer development (Cavenee *et al.* 1983). LOH is shown to create extensive genome variation in *Cryptococcus* hybrids enabling pathogenesis (Li *et al.* 2012). Similarly, LOH events have been associated with the adaptation of *S. cerevisiae* and *Candida albicans* to stressful conditions, such as exposure to antifungals (Selmecki *et al.* 2010; Gerstein *et al.* 2014). Given the role of LOH in evolution and disease, it is useful to understand the extent of LOH over a large number of mitotic divisions. Previous analysis of mitotic LOH events in *S. cerevisiae* that allowed for selection of daughter cells containing the products of mitotic recombination have provided mechanistic insights into mitotic crossovers and gene conversion (Lee *et al.* 2009; Rosen *et al.* 2013; St Charles and Petes 2013; Yim *et al.* 2014). Our study design does not facilitate mechanistic analysis, as the outcome of the DSB repair processes are analyzed after many generations. Instead our study design provides information on the scale of genotypic changes brought about by the accumulated effects of various DSB repair processes over 1140 vegetative divisions (Figure 3). In our study, we have observed: (1) long LOH events extending toward the ends of chromosomes, probably as a result of BIR or mitotic crossovers; (2) small LOH tracts (<1 kb) as a result of local gene conversions; and (3) internal large LOH events that are probably a consequence of repeated mitotic recombination events in response to DNA damage during the propagation of the vegetative lines (Pryszcz *et al.* 2014). Alternatively, they may arise during DSB repair in response to Spo11-mediated DSBs during abortive meiosis. This is supported by the observation that the number and distribution of LOH tracts in V3_57 and V5_57 are distinct from V1_57, V2_57, and V4_57 (Figure 3 and Figure S3, B and C). Extrapolating the spontaneous mitotic crossover rate of 6 × 10^−5^ per division for the right arm of chromosome IV (St Charles and Petes 2013) to the whole genome, only 5–6 crossover/conversion events are expected after 1000 mitotic divisions. The number of long LOH tracts observed in V1_57, V2_57, and V4_57 are consistent with these numbers (Figure 3). But V3_57 and V5_57 have many more long LOH tracts that are also frequently internal. The pattern of LOH in V3_57 and V5_57 would either require a high frequency of DNA damage during vegetative growth or can also be explained, for example, by a double crossover during return to growth. The latter possibility suggests that yeast cells may enter the meiotic program and return to growth during vegetative propagation perhaps in response to nutrient stress. The extensive LOH observed in some of the vegetative lines (presumably due to return to growth) support the idea that LOH is an important tool for the evolution of vegetatively propagating cells by facilitating the fixation of beneficial alleles (Mandegar and Otto 2007). Given the applications of hybrid yeast strains in the industry, knowledge of the scale of genome dynamics in hybrid yeast will also be useful for the design of hybrid yeast genomes (Table S9 in File S2).

### Mutation rates in the hybrid yeast genome

Mutation accumulation experiments have been previously used in a number of model organisms like *S. cerevisiae*, *Drosophila*, and *C. elegans* to estimate spontaneous mutation rates (Denver *et al.* 2000, 2004; Vassilieva *et al.* 2000; Wloch *et al.* 2001; Joseph and Hall 2004; Haag-Liautard *et al.* 2007; Lynch *et al.* 2008; Keightley *et al.* 2009; Nishant *et al.* 2010). Mutation accumulation studies in diploid yeast have been carried out in homozygous backgrounds (Nishant *et al.* 2010; Zhu *et al.* 2014). The use of homozygous lines can affect the mutation process in the cells. For example, fixation of recessive mutator alleles during generation of the homozygous lines can enhance mutation rates, while on the other hand genome rearrangements via ectopic recombination are significantly reduced in homozygous backgrounds compared to heterozygous ones (Montgomery *et al.* 1991; Schrider *et al.* 2013). Given that most organisms have heterozygous genomes, there is a need for accurate estimation of mutation rates in a hybrid context. Our estimate of the average mitotic base mutation rate observed in the S288c/YJM789 hybrid is similar to other homozygous *S. cerevisiae* strains (Lynch *et al.* 2008; Nishant *et al.* 2010; Zhu *et al.* 2014). These observations suggest that the S288c/YJM789 diploid hybrid is not mutagenic. This is relevant, since the S288c/YJM789 hybrid is used extensively for genome-wide mapping of meiotic recombination events (Chen *et al.* 2008; Mancera *et al.* 2008; Oke *et al.* 2014; Krishnaprasad *et al.* 2015).

Previous studies by Yang *et al.* (2015) have shown a 3.4-fold increase in base mutation rates in the F2 progeny of heterozygous F1 *Arabidopsis* plants compared to the homozygous progeny of the selfed plants. A similar 3.4-fold increase in mutation rates was observed in rice heterozygotes over homozygotes (Yang *et al.* 2015). The increased mutagenesis in heterozygous genomes is due to the cis effects of heterozygosity, since homozygous regions within these genomes do not show higher mutation rates. The enhanced mutagenesis is thought to be due to the potential for new mutations during gene conversions associated with meiotic recombination in heterozygous regions (Amos 2010; Yang *et al.* 2015). It may also reflect the enhanced mutagenicity of ssDNA tracts that are generated during meiotic recombination (Yang *et al.* 2008). In order to determine if the heterozygosity of the S288c/YJM789 hybrid contributes to enhanced mutations in meiosis, we need to compare several meiotic lines of the S288c/YJM789 hybrid with isogenic diploid S288c and YJM789 strains, preferably after a single division to avoid the effects of LOH as well as the loss of mutations during intratetrad mating (Nishant *et al.* 2010). Reduced mutation rates were observed in the F3 and F4 selfed *Arabidopsis* plants due to a reduction in heterozygosity (Yang *et al.* 2015). In artificial yeast hybrids, interactions between DNA repair proteins may also play a stronger role in determining the mutation rate. It is possible *S. cerevisiae* hybrid meiotic lines may have elevated mutation rates compared to their homozygous reference strains, due to incompatibility in mismatch repair genes or heterozygosity (Heck *et al.* 2006; Amos 2010; Yang *et al.* 2015).

DNA synthesis at specific loci undergoing mitotic DSB repair in *S. cerevisiae* shows more mutations (Stathern *et al.* 1995). In heterozygous genomes, these effects may be enhanced due to mismatch triggered gene conversions that require additional DNA synthesis similar to the mechanisms proposed for enhanced mutagenesis in heterozygous regions during meiosis (Amos 2010; Yang *et al.* 2015). All the base mutations were observed in the vegetative lines V3_57, V4_57, and V5_57 that showed significant LOH. But a causal link between mutation rate and frequency of LOH in these lines is hindered by the small sample size and lack of tight correlation between the frequency of LOH and the number of mutations (Figure 3). It is also possible that LOH in V3_57 and V5_57 may have been caused by DSBs induced and repaired to a different extent during abortive meiosis followed by completion of the repair during return to growth. In such a scenario, the results would suggest mutagenicity of the meiotic/mitotic recombination process in a heterozygous genome. Mutation rate estimates in other *S. cerevisiae* hybrids will be useful to further consolidate these observations on the mutagenicity of the DSB repair process in a heterozygous context.

## Supplementary Material

Supplemental material is available online at www.g3journal.org/lookup/suppl/doi:10.1534/g3.117.1135/-/DC1.

## References

[bib1] Amos W., 2010 Heterozygosity and mutation rate: evidence for an interaction and its implications: the potential for meiotic gene conversions to influence both mutation rate and distribution. Bioessays 32: 82–90. 19967709 10.1002/bies.200900108

[bib2] Andersen M. P. Nelson Z. W. Hetrick E. D. Gottschling D. E., 2008 A genetic screen for increased loss of heterozygosity in *Saccharomyces cerevisiae*. Genetics 179: 1179–1195. 18562670 10.1534/genetics.108.089250 PMC2475725

[bib3] Antunovics Z. Nguyen H. V. Gaillardin C. Sipiczki M., 2005 Gradual genome stabilisation by progressive reduction of the *Saccharomyces uvarum* genome in an interspecific hybrid with *Saccharomyces cerevisiae*. FEMS Yeast Res. 5: 1141–1150. 15982931 10.1016/j.femsyr.2005.04.008

[bib4] Argueso J. L. Wanat J. Gemici Z. Alani E., 2004 Competing crossover pathways act during meiosis in *Saccharomyces cerevisiae*. Genetics 168: 1805–1816. 15611158 10.1534/genetics.104.032912 PMC1448724

[bib5] Argueso J. L. Carazzolle M. F. Mieczkowski P. A. Duarte F. M. Netto O. V., 2009 Genome structure of a *Saccharomyces cerevisiae* strain widely used in bioethanol production. Genome Res. 19: 2258–2270. 19812109 10.1101/gr.091777.109 PMC2792172

[bib6] Barbera M. A. Petes T. D., 2006 Selection and analysis of spontaneous reciprocal mitotic cross-overs in *Saccharomyces cerevisiae*. Proc. Natl. Acad. Sci. USA 103: 12819–12824. 16908833 10.1073/pnas.0605778103 PMC1550776

[bib7] Bergelson J. Roux F., 2010 Towards identifying genes underlying ecologically relevant traits in *Arabidopsis thaliana*. Nat. Rev. Genet. 11: 867–879. 21085205 10.1038/nrg2896

[bib8] Borneman A. R. Desany B. A. Riches D. Affourtit J. P. Forgan A. H., 2011 Whole-genome comparison reveals novel genetic elements that characterize the genome of industrial strains of *Saccharomyces cerevisiae*. PLoS Genet. 7: e1001287. 21304888 10.1371/journal.pgen.1001287 PMC3033381

[bib9] Burke M. K. Dunham J. P. Shahrestani P. Thornton K. R. Rose M. R., 2010 Genome-wide analysis of a long-term evolution experiment with *Drosophila*. Nature 467: 587–590. 20844486 10.1038/nature09352

[bib10] Cavenee W. K. Dryja T. P. Phillips R. A. Benedict W. F. Godbout R., 1983 Expression of recessive alleles by chromosomal mechanisms in retinoblastoma. Nature 305: 779–784. 6633649 10.1038/305779a0

[bib11] Chakraborty U. Alani E., 2016 Understanding how mismatch repair proteins participate in the repair/anti-recombination decision. FEMS Yeast Res. 16: fow071. 27573382 10.1093/femsyr/fow071 PMC5976031

[bib12] Chen S. Y. Tsubouchi T. Rockmill B. Sandler J. S. Richards D. R., 2008 Global analysis of the meiotic crossover landscape. Dev. Cell 15: 401–415. 18691940 10.1016/j.devcel.2008.07.006 PMC2628562

[bib13] Cubillos F. A. Billi E. Zorgo E. Parts L. Fargier P., 2011 Assessing the complex architecture of polygenic traits in diverged yeast populations. Mol. Ecol. 20: 1401–1413. 21261765 10.1111/j.1365-294X.2011.05005.x

[bib14] Dayani Y. Simchen G. Lichten M., 2011 Meiotic recombination intermediates are resolved with minimal crossover formation during return-to-growth, an analogue of the mitotic cell cycle. PLoS Genet. 7: e1002083. 21637791 10.1371/journal.pgen.1002083 PMC3102748

[bib15] Denver D. R. Morris K. Lynch M. Vassilieva L. L. Thomas W. K., 2000 High direct estimate of the mutation rate in the mitochondrial genome of *Caenorhabditis elegans*. Science 289: 2342–2344. 11009418 10.1126/science.289.5488.2342

[bib16] Denver D. R. Morris K. Lynch M. Thomas W. K., 2004 High mutation rate and predominance of insertions in the *Caenorhabditis elegans* nuclear genome. Nature 430: 679–682. 15295601 10.1038/nature02697

[bib17] Dunham M. J. Badrane H. Ferea T. Adams J. Brown P. O., 2002 Characteristic genome rearrangements in experimental evolution of *Saccharomyces cerevisiae*. Proc. Natl. Acad. Sci. USA 99: 16144–16149. 12446845 10.1073/pnas.242624799 PMC138579

[bib18] Dunn B. Paulish T. Stanbery A. Piotrowski J. Koniges G., 2013 Recurrent rearrangement during adaptive evolution in an interspecific yeast hybrid suggests a model for rapid introgression. PLoS Genet. 9: e1003366. 23555283 10.1371/journal.pgen.1003366 PMC3605161

[bib19] Flint J. Mott R., 2001 Finding the molecular basis of quantitative traits: successes and pitfalls. Nat. Rev. Genet. 2: 437–445. 11389460 10.1038/35076585

[bib20] Flynn J. M. Chain F. J. Schoen D. J. Cristescu M. E., 2017 Spontaneous mutation accumulation in *Daphnia pulex* in selection-free *vs.* competitive environments. Mol. Biol. Evol. 34: 160–173. 27777284 10.1093/molbev/msw234

[bib21] Gerstein A. C. Kuzmin A. Otto S. P., 2014 Loss-of-heterozygosity facilitates passage through Haldane’s sieve for *Saccharomyces cerevisiae* undergoing adaptation. Nat. Commun. 5: 3819. 24804896 10.1038/ncomms4819

[bib22] Greig D. Louis E. J. Borts R. H. Travisano M., 2002 Hybrid speciation in experimental populations of yeast. Science 298: 1773–1775. 12459586 10.1126/science.1076374

[bib23] Gresham D. Ruderfer D. M. Pratt S. C. Schacherer J. Dunham M. J., 2006 Genome-wide detection of polymorphisms at nucleotide resolution with a single DNA microarray. Science 311: 1932–1936. 16527929 10.1126/science.1123726

[bib24] Haag-Liautard C. Dorris M. Maside X. Macaskill S. Halligan D. L., 2007 Direct estimation of per nucleotide and genomic deleterious mutation rates in *Drosophila*. Nature 445: 82–85. 17203060 10.1038/nature05388

[bib25] Halligan D. L. Keightley P. D., 2009 Spontaneous mutation accumulation studies in evolutionary genetics. Annu. Rev. Ecol. Evol. Syst. 40: 151–172.

[bib26] Hansche P. E. Beres V. Lange P., 1978 Gene duplication in *Saccharomyces cerevisiae*. Genetics 88: 673–687. 348562 PMC1213812

[bib27] Heck J. A. Argueso J. L. Gemici Z. Reeves R. G. Bernard A., 2006 Negative epistasis between natural variants of the *Saccharomyces cerevisiae* MLH1 and PMS1 genes results in a defect in mismatch repair. Proc. Natl. Acad. Sci. USA 103: 3256–3261. 16492773 10.1073/pnas.0510998103 PMC1413905

[bib28] Joseph S. B. Hall D. W., 2004 Spontaneous mutations in diploid *Saccharomyces cerevisiae*: more beneficial than expected. Genetics 168: 1817–1825. 15611159 10.1534/genetics.104.033761 PMC1448740

[bib29] Keightley P. D. Trivedi U. Thomson M. Oliver F. Kumar S., 2009 Analysis of the genome sequences of three *Drosophila melanogaster* spontaneous mutation accumulation lines. Genome Res. 19: 1195–1201. 19439516 10.1101/gr.091231.109 PMC2704435

[bib30] Kelly A. C. Shewmaker F. P. Kryndushkin D. Wickner R. B., 2012 Sex, prions, and plasmids in yeast. Proc. Natl. Acad. Sci. USA 109: E2683–E2690. 22949655 10.1073/pnas.1213449109 PMC3479589

[bib31] Knop M., 2006 Evolution of the hemiascomycete yeasts: on life styles and the importance of inbreeding. Bioessays 28: 696–708. 16929561 10.1002/bies.20435

[bib32] Krishnaprasad G. N. Anand M. T. Lin G. Tekkedil M. M. Steinmetz L. M., 2015 Variation in crossover frequencies perturb crossover assurance without affecting meiotic chromosome segregation in *Saccharomyces cerevisiae*. Genetics 199: 399–412. 25467183 10.1534/genetics.114.172320 PMC4317650

[bib33] Langmead B. Salzberg S. L., 2012 Fast gapped-read alignment with Bowtie 2. Nat. Methods 9: 357–359. 22388286 10.1038/nmeth.1923 PMC3322381

[bib34] Laureau R. Loeillet S. Salinas F. Bergstrom A. Legoix-Ne P., 2016 Extensive recombination of a yeast diploid hybrid through meiotic reversion. PLoS Genet. 12: e1005781. 26828862 10.1371/journal.pgen.1005781 PMC4734685

[bib35] Lee H. Popodi E. Tang H. Foster P. L., 2012 Rate and molecular spectrum of spontaneous mutations in the bacterium *Escherichia coli* as determined by whole-genome sequencing. Proc. Natl. Acad. Sci. USA 109: E2774–E2783. 22991466 10.1073/pnas.1210309109 PMC3478608

[bib36] Lee P. S. Greenwell P. W. Dominska M. Gawel M. Hamilton M., 2009 A fine-structure map of spontaneous mitotic crossovers in the yeast *Saccharomyces cerevisiae*. PLoS Genet. 5: e1000410. 19282969 10.1371/journal.pgen.1000410 PMC2646836

[bib37] Li W. Averette A. F. Desnos-Ollivier M. Ni M. Dromer F., 2012 Genetic diversity and genomic plasticity of *Cryptococcus neoformans* AD hybrid strains. G3 2: 83–97. 22384385 10.1534/g3.111.001255 PMC3276195

[bib38] Liti G. Barton D. B. Louis E. J., 2006 Sequence diversity, reproductive isolation and species concepts in *Saccharomyces*. Genetics 174: 839–850. 16951060 10.1534/genetics.106.062166 PMC1602076

[bib39] Liu Y. Gaines W. A. Callender T. Busygina V. Oke A., 2014 Down-regulation of Rad51 activity during meiosis in yeast prevents competition with Dmc1 for repair of double-strand breaks. PLoS Genet. 10: e1004005. 24465215 10.1371/journal.pgen.1004005 PMC3900393

[bib40] Lynch M. Sung W. Morris K. Coffey N. Landry C. R., 2008 A genome-wide view of the spectrum of spontaneous mutations in yeast. Proc. Natl. Acad. Sci. USA 105: 9272–9277. 18583475 10.1073/pnas.0803466105 PMC2453693

[bib41] Magwene P. M., 2014 Revisiting Mortimer’s genome renewal hypothesis: heterozygosity, homothallism, and the potential for adaptation in yeast. Adv. Exp. Med. Biol. 781: 37–48. 24277294 10.1007/978-94-007-7347-9_3 PMC4096854

[bib42] Magwene P. M. Kayıkçı Ö. Granek J. A. Reininga J. M. Scholl Z., 2011 Outcrossing, mitotic recombination, and life-history trade-offs shape genome evolution in *Saccharomyces cerevisiae*. Proc. Natl. Acad. Sci. USA 108: 1987–1992. 21245305 10.1073/pnas.1012544108 PMC3033294

[bib43] Mancera E. Bourgon R. Brozzi A. Huber W. Steinmetz L. M., 2008 High-resolution mapping of meiotic crossovers and non-crossovers in yeast. Nature 454: 479–485. 18615017 10.1038/nature07135 PMC2780006

[bib44] Mandegar M. A. Otto S. P., 2007 Mitotic recombination counteracts the benefits of genetic segregation. Proc. Biol. Sci. 274: 1301–1307. 17360283 10.1098/rspb.2007.0056 PMC2176173

[bib45] Martini E. Borde V. Legendre M. Audic S. Regnault B., 2011 Genome-wide analysis of heteroduplex DNA in mismatch repair-deficient yeast cells reveals novel properties of meiotic recombination pathways. PLoS Genet. 7: e1002305. 21980306 10.1371/journal.pgen.1002305 PMC3183076

[bib46] McCusker J. H. Clemons K. V. Stevens D. A. Davis R. W., 1994 Genetic characterization of pathogenic *Saccharomyces cerevisiae* isolates. Genetics 136: 1261–1269. 8013903 10.1093/genetics/136.4.1261 PMC1205906

[bib47] Montgomery E. A. Huang S. M. Langley C. H. Judd B. H., 1991 Chromosome rearrangement by ectopic recombination in *Drosophila melanogaster*: genome structure and evolution. Genetics 129: 1085–1098. 1783293 10.1093/genetics/129.4.1085 PMC1204773

[bib48] Morales L. Dujon B., 2012 Evolutionary role of interspecies hybridization and genetic exchanges in yeasts. Microbiol. Mol. Biol. Rev. 76: 721–739. 23204364 10.1128/MMBR.00022-12 PMC3510521

[bib49] Mortimer R. K. Romano P. Suzzi G. Polsinelli M., 1994 Genome renewal: a new phenomenon revealed from a genetic study of 43 strains of *Saccharomyces cerevisiae* derived from natural fermentation of grape musts. Yeast 10: 1543–1552. 7725789 10.1002/yea.320101203

[bib50] Nishant K. T. Singh N. D. Alani E., 2009 Genomic mutation rates: what high-throughput methods can tell us. Bioessays 31: 912–920. 19644920 10.1002/bies.200900017 PMC2952423

[bib51] Nishant K. T. Wei W. Mancera E. Argueso J. L. Schlattl A., 2010 The baker’s yeast diploid genome is remarkably stable in vegetative growth and meiosis. PLoS Genet. 6: e1001109. 20838597 10.1371/journal.pgen.1001109 PMC2936533

[bib52] Oke A. Anderson C. M. Yam P. Fung J. C., 2014 Controlling meiotic recombinational repair – specifying the roles of ZMMs, Sgs1 and Mus81/Mms4 in crossover formation. PLoS Genet. 10: e1004690. 25329811 10.1371/journal.pgen.1004690 PMC4199502

[bib53] Palmer S. Schildkraut E. Lazarin R. Nguyen J. Nickoloff J. A., 2003 Gene conversion tracts in *Saccharomyces cerevisiae* can be extremely short and highly directional. Nucleic Acids Res. 31: 1164–1173. 12582235 10.1093/nar/gkg219 PMC150237

[bib54] Paques F. Haber J. E., 1999 Multiple pathways of recombination induced by double-strand breaks in *Saccharomyces cerevisiae*. Microbiol. Mol. Biol. Rev. 63: 349–404. 10357855 10.1128/mmbr.63.2.349-404.1999 PMC98970

[bib55] Pryszcz L. P. Nemeth T. Gacser A. Gabaldon T., 2014 Genome comparison of *Candida orthopsilosis* clinical strains reveals the existence of hybrids between two distinct subspecies. Genome Biol. Evol. 6: 1069–1078. 24747362 10.1093/gbe/evu082 PMC4040990

[bib56] Qi J. Wijeratne A. J. Tomsho L. P. Hu Y. Schuster S. C., 2009 Characterization of meiotic crossovers and gene conversion by whole-genome sequencing in *Saccharomyces cerevisiae*. BMC Genomics 10: 475. 19832984 10.1186/1471-2164-10-475 PMC2770529

[bib57] Querol A. Bond U., 2009 The complex and dynamic genomes of industrial yeasts. FEMS Microbiol. Lett. 293: 1–10. 19175410 10.1111/j.1574-6968.2008.01480.x

[bib58] Rose M. D. Winston F. M. Heiter P., 1990 *Methods in Yeast Genetics: A Laboratory Course Manual*. Cold Spring Harbor Laboratory Press, New York.

[bib59] Rosen D. M. Younkin E. M. Miller S. D. Casper A. M., 2013 Fragile site instability in *Saccharomyces cerevisiae* causes loss of heterozygosity by mitotic crossovers and break-induced replication. PLoS Genet. 9: e1003817. 24068975 10.1371/journal.pgen.1003817 PMC3778018

[bib60] Ruderfer D. M. Pratt S. C. Seidel H. S. Kruglyak L., 2006 Population genomic analysis of outcrossing and recombination in yeast. Nat. Genet. 38: 1077–1081. 16892060 10.1038/ng1859

[bib61] Schrider D. R. Houle D. Lynch M. Hahn M. W., 2013 Rates and genomic consequences of spontaneous mutational events in *Drosophila melanogaster*. Genetics 194: 937–954. 23733788 10.1534/genetics.113.151670 PMC3730921

[bib62] Selmecki A. Forche A. Berman J., 2010 Genomic plasticity of the human fungal pathogen *Candida albicans*. Eukaryot. Cell 9: 991–1008. 20495058 10.1128/EC.00060-10 PMC2901674

[bib63] St Charles J. Petes T. D., 2013 High-resolution mapping of spontaneous mitotic recombination hotspots on the 1.1 Mb arm of yeast chromosome IV. PLoS Genet. 9: e1003434. 23593029 10.1371/journal.pgen.1003434 PMC3616911

[bib64] Steinmetz L. M. Sinha H. Richards D. R. Spiegelman J. I. Oefner P. J., 2002 Dissecting the architecture of a quantitative trait locus in yeast. Nature 416: 326–330. 11907579 10.1038/416326a

[bib65] Stelkens R. Brockhurst M. Hurst G. Miller E. Greig D., 2014 The effect of hybrid transgression on environmental tolerance in experimental yeast crosses. J. Evol. Biol. 27: 2507–2519. 25262771 10.1111/jeb.12494

[bib66] Strathern J. N. Shafer B. K. McGill C. B., 1995 DNA synthesis errors associated with double-strand-break repair. Genetics 140: 965–972. 7672595 10.1093/genetics/140.3.965 PMC1206680

[bib67] Tang S. Okashah R. A. Knapp S. J. Arnold M. L. Martin N. H., 2010 Transmission ratio distortion results in asymmetric introgression in Louisiana Iris. BMC Plant Biol. 10: 48. 20298609 10.1186/1471-2229-10-48 PMC2923522

[bib68] Vassilieva L. L. Hook A. M. Lynch M., 2000 The fitness effects of spontaneous mutations in *Caenorhabditis elegans*. Evolution 54: 1234–1246. 11005291 10.1111/j.0014-3820.2000.tb00557.x

[bib69] Wei W. McCusker J. H. Hyman R. W. Jones T. Ning Y., 2007 Genome sequencing and comparative analysis of *Saccharomyces cerevisiae* strain YJM789. Proc. Natl. Acad. Sci. USA 104: 12825–12830. 17652520 10.1073/pnas.0701291104 PMC1933262

[bib70] Wloch D. M. Szafraniec K. Borts R. H. Korona R., 2001 Direct estimate of the mutation rate and the distribution of fitness effects in the yeast *Saccharomyces cerevisiae*. Genetics 159: 441–452. 11606524 10.1093/genetics/159.2.441 PMC1461830

[bib71] Wolfe K. H., 2015 Origin of the yeast whole-genome duplication. PLoS Biol. 13: e1002221. 26252643 10.1371/journal.pbio.1002221 PMC4529243

[bib72] Yang S. Wang L. Huang J. Zhang X. Yuan Y., 2015 Parent-progeny sequencing indicates higher mutation rates in heterozygotes. Nature 523: 463–467. 26176923 10.1038/nature14649

[bib73] Yang Y. Sterling J. Storici F. Resnick M. A. Gordenin D. A., 2008 Hypermutability of damaged single-strand DNA formed at double-strand breaks and uncapped telomeres in yeast *Saccharomyces cerevisiae*. PLoS Genet. 4: e1000264. 19023402 10.1371/journal.pgen.1000264 PMC2577886

[bib74] Yim E. O’Connell K. E. Charles J. S. Petes T. D., 2014 High-resolution mapping of two types of spontaneous mitotic gene conversion events in *Saccharomyces cerevisiae*. Genetics 198: 181–192. 24990991 10.1534/genetics.114.167395 PMC4174931

[bib75] Yin Y. Petes T. D., 2013 Genome-wide high-resolution mapping of UV-induced mitotic recombination events in *Saccharomyces cerevisiae*. PLoS Genet. 9: e1003894. 24204306 10.1371/journal.pgen.1003894 PMC3814309

[bib76] Zhu Y. O. Siegal M. L. Hall D. W. Petrov D. A., 2014 Precise estimates of mutation rate and spectrum in yeast. Proc. Natl. Acad. Sci. USA 111: E2310–E2318. 24847077 10.1073/pnas.1323011111 PMC4050626

