# Supplementary material for: Genome Dynamics of Hybrid Saccharomyces cerevisiae During Vegetative and Meiotic Divisions
Source: G3 (Bethesda). 2017 Sep 15;7(11):3669–79. doi: 10.1534/g3.117.1135 (PMC5677154; doi:10.1534/g3.117.1135)
Supplement: Supplementary file 1 [file 3669FigureS1.pptx]

## Slide 1
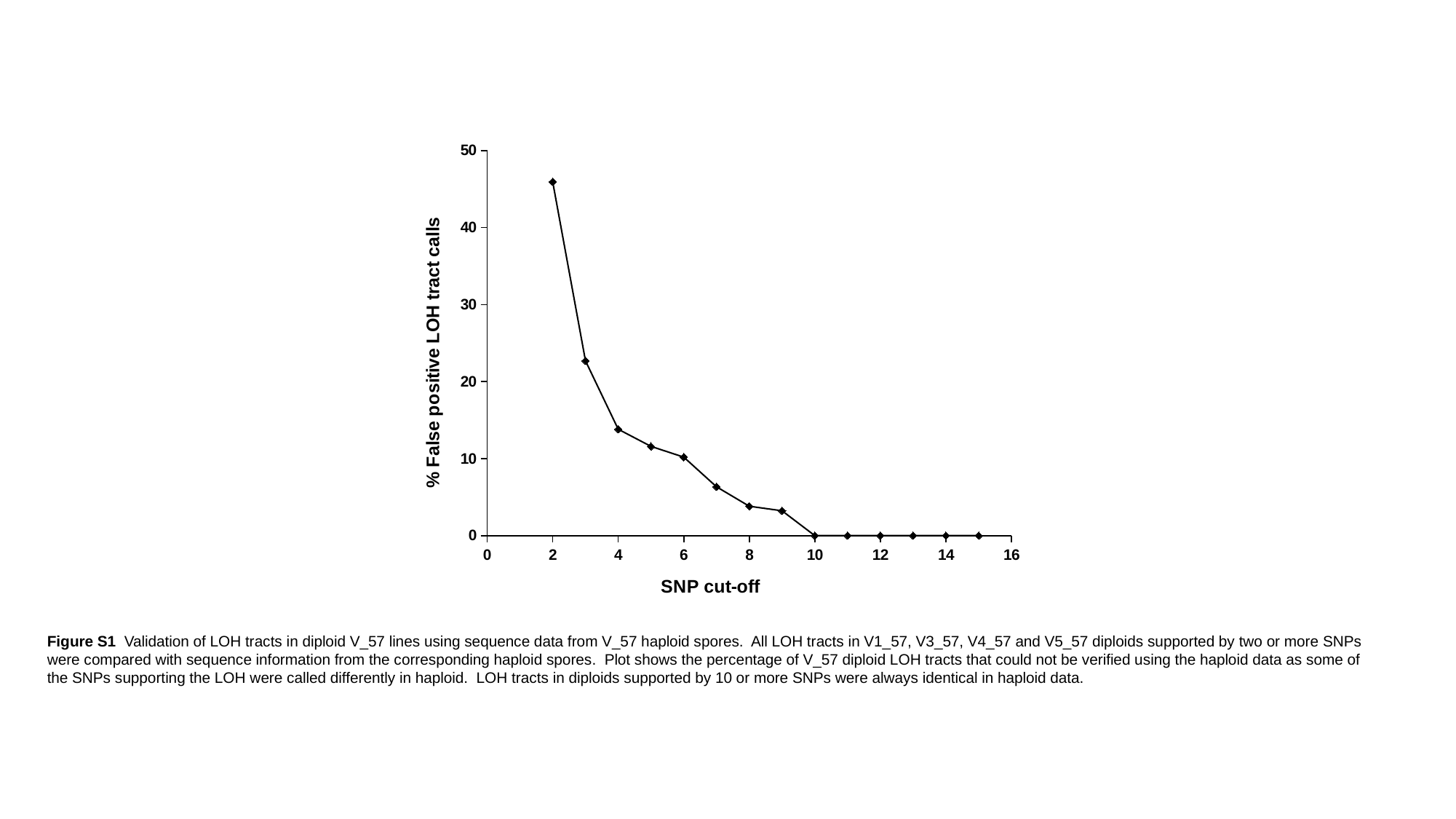

### Chart
| Category | % False positive LOH tract calls |
|---|---|Figure S1 Validation of LOH tracts in diploid V_57 lines using sequence data from V_57 haploid spores. All LOH tracts in V1_57, V3_57, V4_57 and V5_57 diploids supported by two or more SNPs were compared with sequence information from the corresponding haploid spores. Plot shows the percentage of V_57 diploid LOH tracts that could not be verified using the haploid data as some of the SNPs supporting the LOH were called differently in haploid. LOH tracts in diploids supported by 10 or more SNPs were always identical in haploid data.
