# Supplementary material for: Genome Dynamics of Hybrid Saccharomyces cerevisiae During Vegetative and Meiotic Divisions
Source: G3 (Bethesda). 2017 Sep 15;7(11):3669–79. doi: 10.1534/g3.117.1135 (PMC5677154; doi:10.1534/g3.117.1135)
Supplement: Supplementary file 2 [file 3669FigureS2.pptx]

## Slide 1
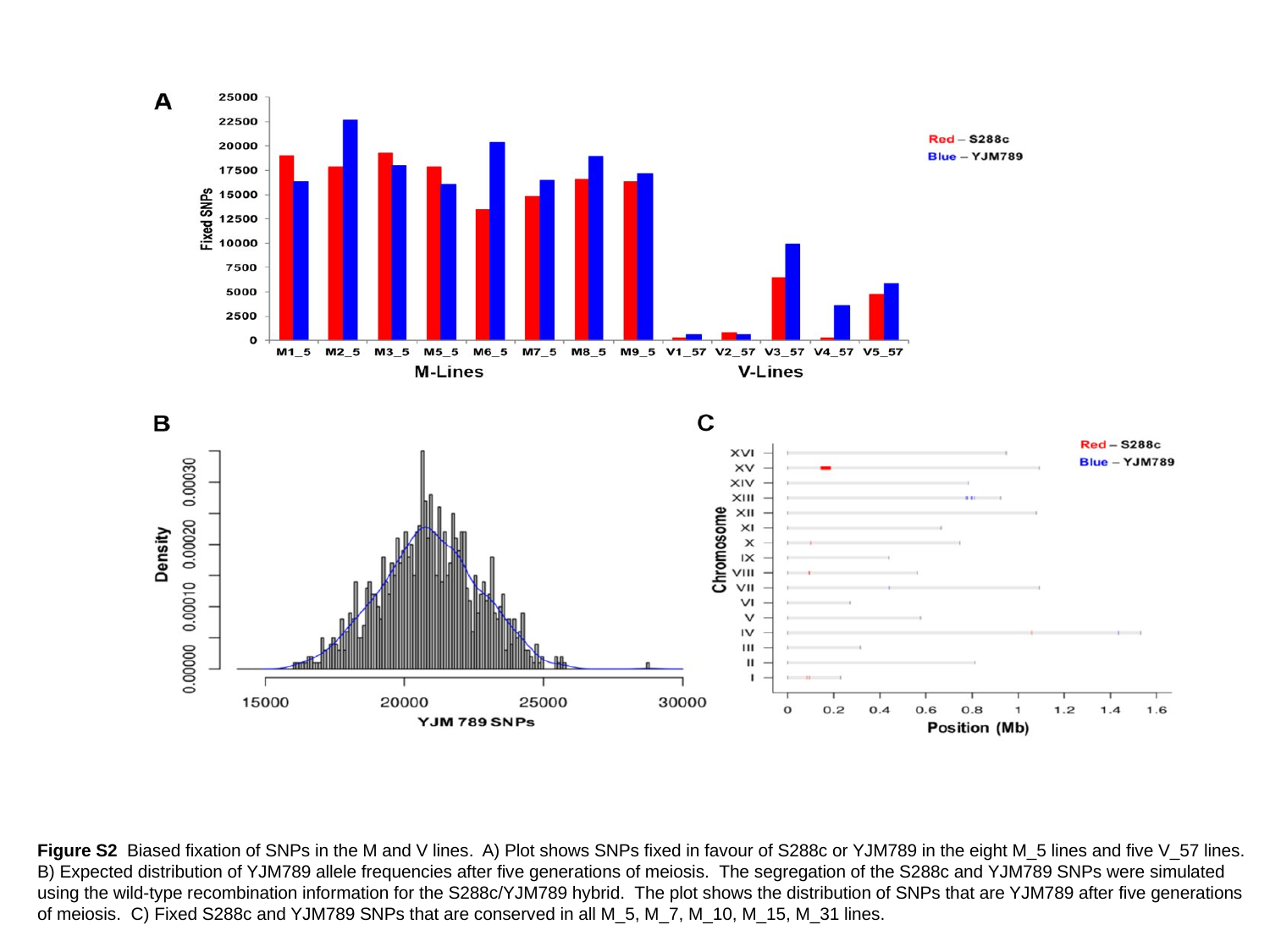

Figure S2 Biased fixation of SNPs in the M and V lines. A) Plot shows SNPs fixed in favour of S288c or YJM789 in the eight M_5 lines and five V_57 lines. B) Expected distribution of YJM789 allele frequencies after five generations of meiosis. The segregation of the S288c and YJM789 SNPs were simulated using the wild-type recombination information for the S288c/YJM789 hybrid. The plot shows the distribution of SNPs that are YJM789 after five generations of meiosis. C) Fixed S288c and YJM789 SNPs that are conserved in all M_5, M_7, M_10, M_15, M_31 lines.
