# Supplementary material for: Genome Dynamics of Hybrid Saccharomyces cerevisiae During Vegetative and Meiotic Divisions
Source: G3 (Bethesda). 2017 Sep 15;7(11):3669–79. doi: 10.1534/g3.117.1135 (PMC5677154; doi:10.1534/g3.117.1135)
Supplement: Supplementary file 3 [file 3669FigureS3.pptx]

## Slide 1
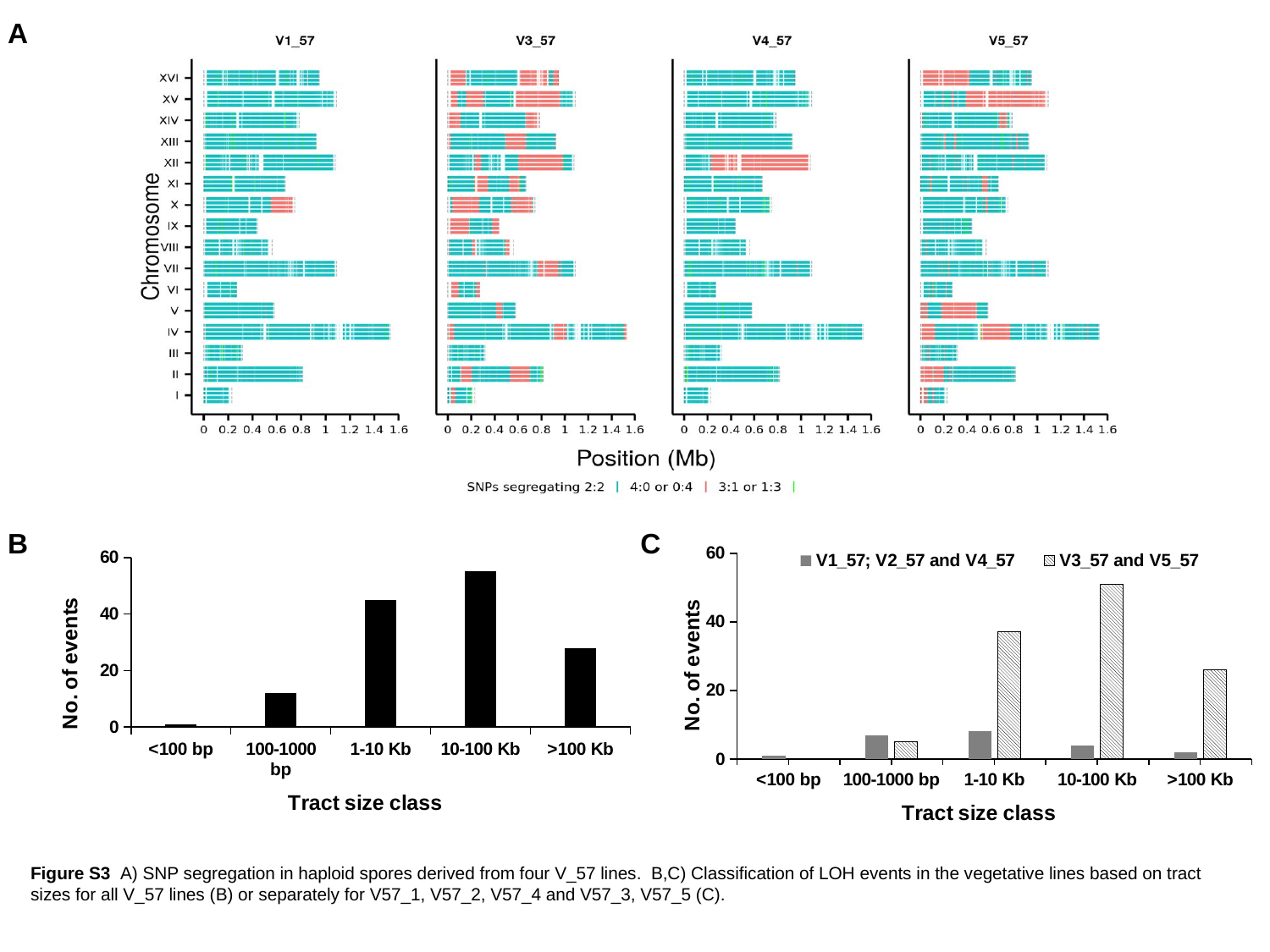

A
B
C
### Chart
| Category | V1_57; V2_57 and V4_57 | V3_57 and V5_57 |
|---|---|---|
| <100 bp | 1.0 | 0.0 |
| 100-1000 bp | 7.0 | 5.0 |
| 1-10 Kb | 8.0 | 37.0 |
| 10-100 Kb | 4.0 | 51.0 |
| >100 Kb | 2.0 | 26.0 |
### Chart
| Category | No of events |
|---|---|
| <100 bp | 1.0 |
| 100-1000 bp | 12.0 |
| 1-10 Kb | 45.0 |
| 10-100 Kb | 55.0 |
| >100 Kb | 28.0 |Figure S3 A) SNP segregation in haploid spores derived from four V_57 lines. B,C) Classification of LOH events in the vegetative lines based on tract sizes for all V_57 lines (B) or separately for V57_1, V57_2, V57_4 and V57_3, V57_5 (C).
