# Supplementary material for: Genome Dynamics of Hybrid Saccharomyces cerevisiae During Vegetative and Meiotic Divisions
Source: G3 (Bethesda). 2017 Sep 15;7(11):3669–79. doi: 10.1534/g3.117.1135 (PMC5677154; doi:10.1534/g3.117.1135)
Supplement: Supplementary file 4 [file 3669FigureS4.pdf]

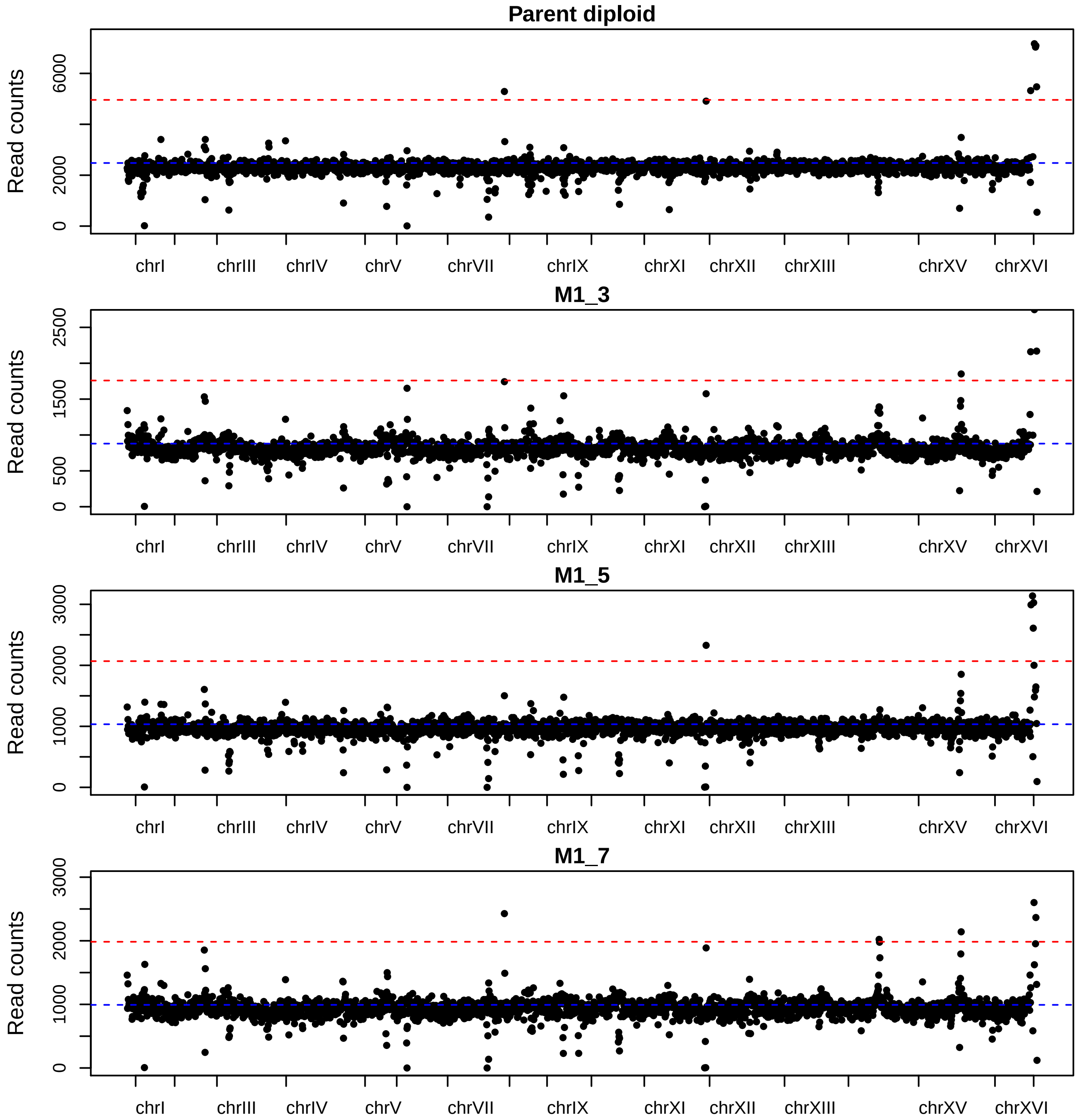
Parent diploid
M1_3
M1_5
M1_7
Read counts
0
2000
6000
500
1500
2500
1000
3000
chrI
chrIII
chrIV
chrV
chrVII
chrIX
chrXI
chrXII
chrXIII
chrXV
chrXVI

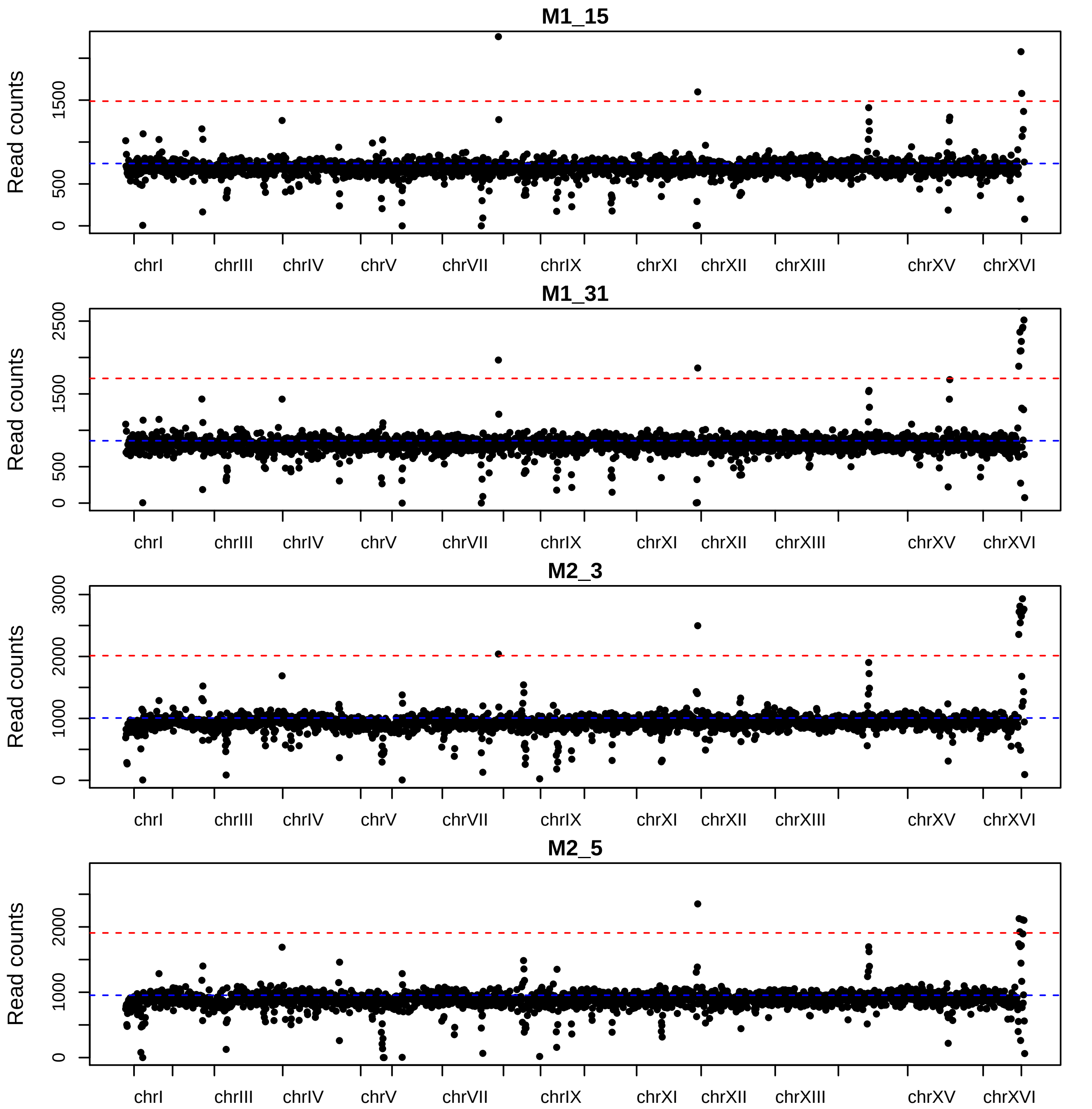
M1_15
Read counts
0
500
1500
chrI
chrIII
chrIV
chrV
chrVII
chrIX
chrXI
chrXII
chrXIII
chrXV
chrXVI
M1_31
Read counts
0
500
1500
2500
chrI
chrIII
chrIV
chrV
chrVII
chrIX
chrXI
chrXII
chrXIII
chrXV
chrXVI
M2_3
Read counts
0
1000
2000
3000
chrI
chrIII
chrIV
chrV
chrVII
chrIX
chrXI
chrXII
chrXIII
chrXV
chrXVI
M2_5
Read counts
0
1000
2000
chrI
chrIII
chrIV
chrV
chrVII
chrIX
chrXI
chrXII
chrXIII
chrXV
chrXVI

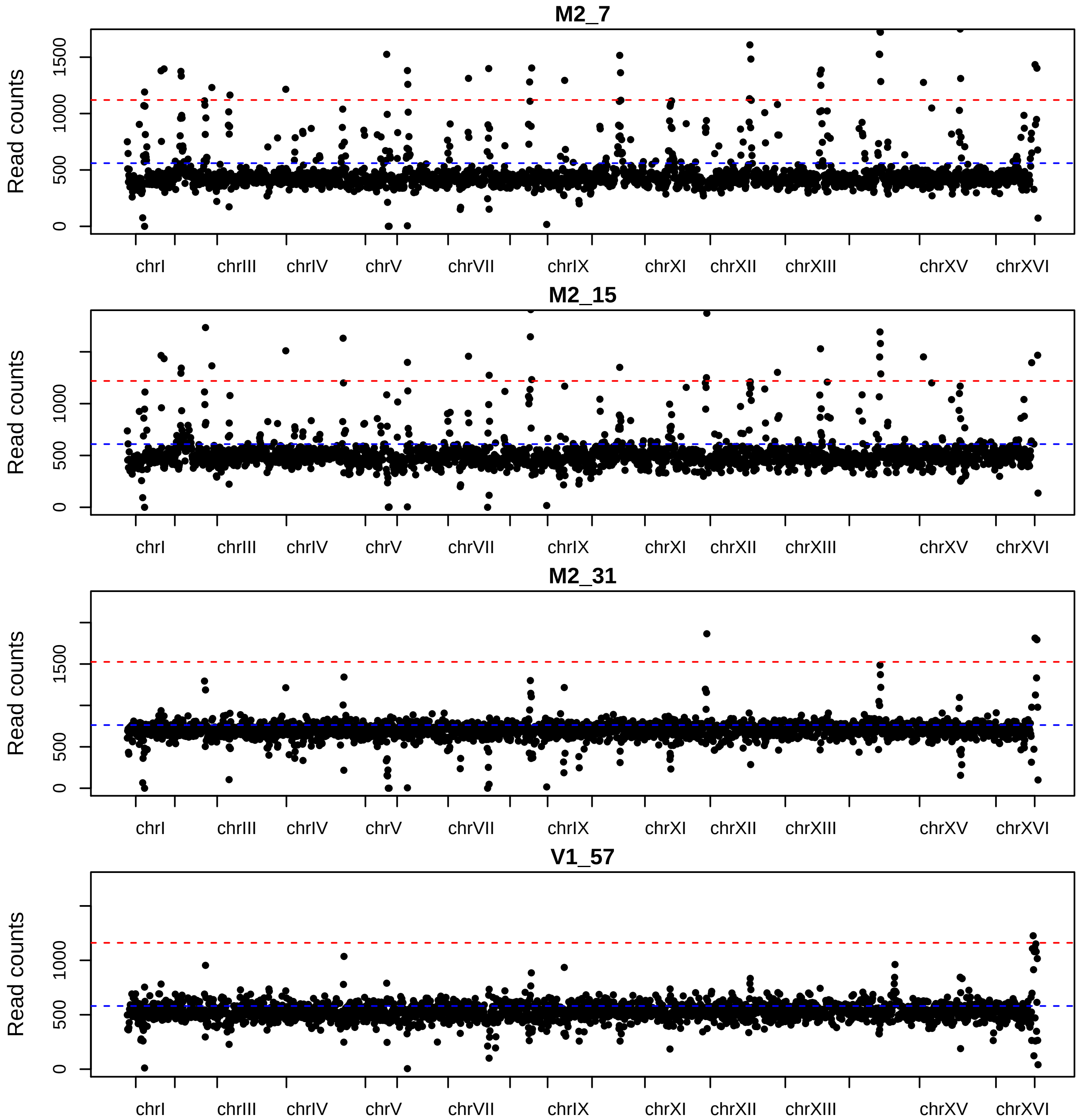
M2_7
M2_15
M2_31
V1_57
Read counts
0
500
1000
1500
chrI
chrIII
chrIV
chrV
chrVII
chrIX
chrXI
chrXII
chrXIII
chrXV
chrXVI

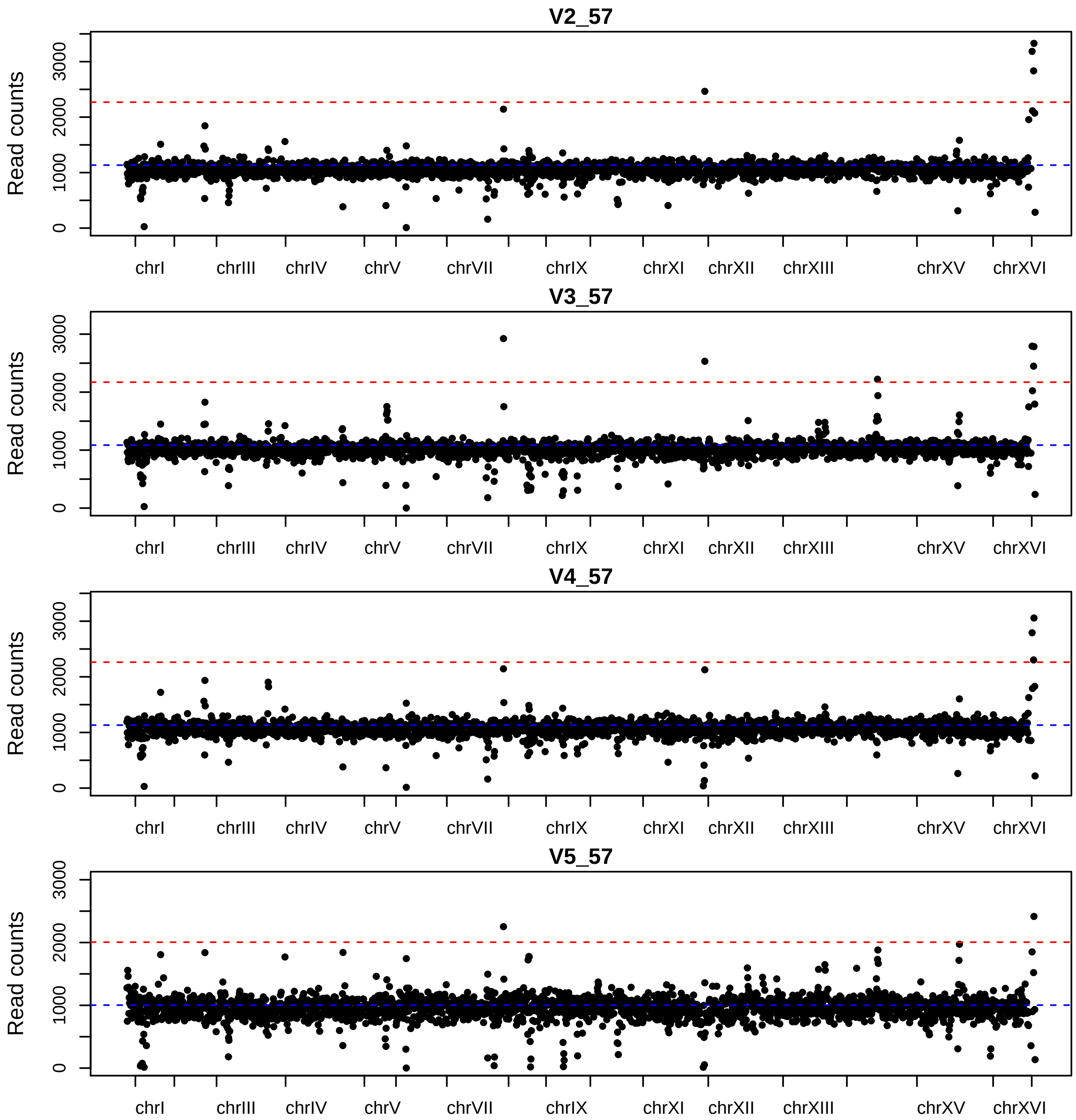

V2_57
V3_57
V4_57
V5_57
Read counts
0
1000
2000
3000
chrI
chrIII
chrIV
chrV
chrVII
chrIX
chrXI
chrXII
chrXIII
chrXV
chrXVI

Figure S4 Absence of chromosomal aneuploidy in the M lines 1 and 2 and the five vegetative lines. Each dot indicates the total read count in 5 kb bins. The blue and red lines show the average and 2X average read count.
