# Supplementary material for: Genome Dynamics of Hybrid Saccharomyces cerevisiae During Vegetative and Meiotic Divisions
Source: G3 (Bethesda). 2017 Sep 15;7(11):3669–79. doi: 10.1534/g3.117.1135 (PMC5677154; doi:10.1534/g3.117.1135)
Supplement: Supplementary file 5 [file 3669FigureS5.pptx]

## Slide 1
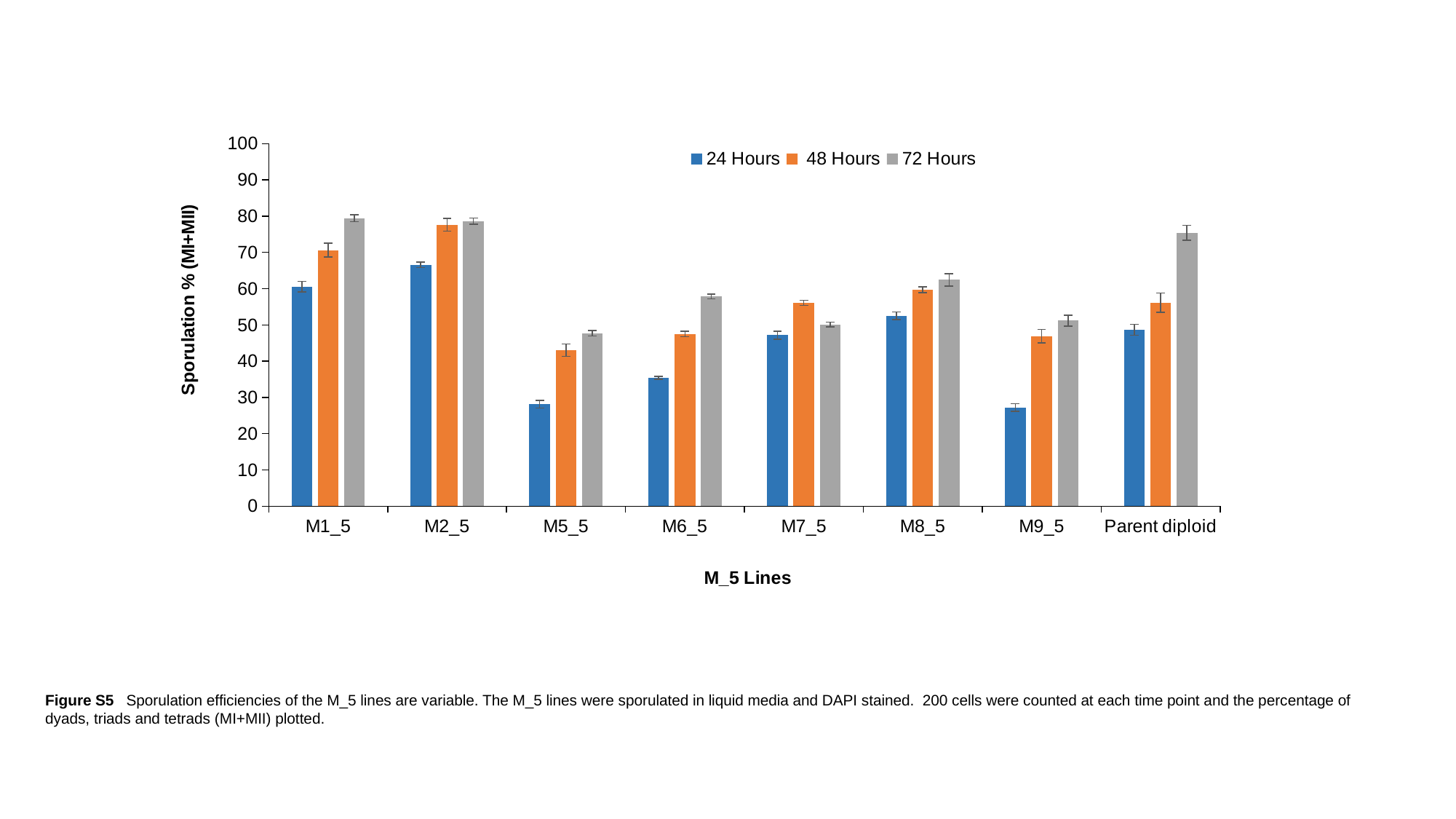

### Chart
| Category | 24 Hours | 48 Hours | 72 Hours |
|---|---|---|---|
| M1_5 | 60.549 | 70.63866666666667 | 79.41166666666668 |
| M2_5 | 66.57533333333333 | 77.61733333333332 | 78.67433333333334 |
| M5_5 | 28.115 | 43.05033333333333 | 47.69766666666667 |
| M6_5 | 35.37366666666666 | 47.55333333333333 | 57.85633333333334 |
| M7_5 | 47.18466666666666 | 56.10533333333333 | 50.15133333333333 |
| M8_5 | 52.53233333333332 | 59.682 | 62.44366666666667 |
| M9_5 | 27.19833333333333 | 46.893 | 51.17166666666666 |
| Parent diploid | 48.646 | 56.13733333333334 | 75.38133333333333 |Figure S5 Sporulation efficiencies of the M_5 lines are variable. The M_5 lines were sporulated in liquid media and DAPI stained. 200 cells were counted at each time point and the percentage of dyads, triads and tetrads (MI+MII) plotted.
