# Supplementary material for: Genome Dynamics of Hybrid Saccharomyces cerevisiae During Vegetative and Meiotic Divisions
Source: G3 (Bethesda). 2017 Sep 15;7(11):3669–79. doi: 10.1534/g3.117.1135 (PMC5677154; doi:10.1534/g3.117.1135)
Supplement: Supplementary file 13 [file 3669FileS1.pdf]

File S1

Comparison of genotype data from diploid and haploid sequencing of the M lines

We compared the number of heterozygous markers that can be detected in the M lines from analysis of the diploid colonies versus haploid segregants. In our experiment, the diploid colony arising from the tetrad represents a mixture of genotypes representing all possible spore mating combinations within the tetrad. Part of this diploid colony is frozen as the stock and part of it is continued to the next round of meiosis. So when the diploid colony is sporulated and a single tetrad is sequenced as haploid spores, we are sampling a portion of the heterozygous alleles in the diploid. As a consequence when we trace the lineage of the SNPs using spore data, SNPs that appear fixed in a particular generation of meiosis, may appear to be heterozygous or fixed to the opposite genotype in subsequent generations (Table S3). This problem affects a small number of the markers and as predicted, when we analysed sites that showed switching of fixed YJM789 and S288c alleles between M1_5 and M1_7, they were all heterozygous in M1_3 (Figure S6).

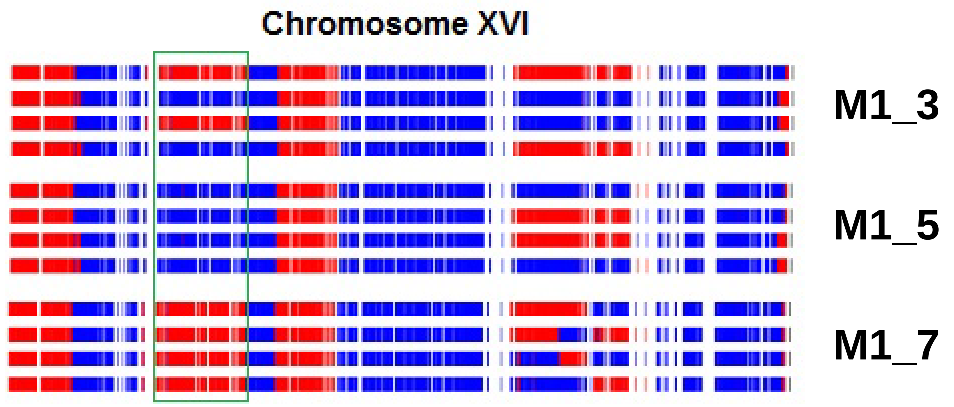

Figure S6 An example (green box) of heterozygous SNPs in M1_3 that fix towards YJM789 (Blue) in M1_5 and S288c (Red) in M1_7 when spore sequencing data is used. SNPs that are fixed did not show such switches.

This problem does not occur when homozygous lines are used (NISHANT *et al.* 2010). Comparison of heterozygous sites in diploid versus spore data for the two M lines (1 and 2) showed excess of heterozygous segregating sites in the diploid data as expected from the experimental design (Table S8).

Table S8 Comparison of heterozygous SNP counts in the M lines based on sequencing of the diploid strain and it's four haploid spores.

Line	Heterozygous segregating sites	
	Diploid sequencing (1:1)	Haploid sequencing (2:2)
M line common SNPs	46281	43122
M1_3	14890	10281
M2_3	15655	11084
M1_5	10878	7047
M2_5	5785	5524
M1_7	4688	3310
M2_7	5800	2429
M1_10	1453	1146
M2_10	1802	1567
M1_15	995	677
M2_15	1401	1104
M1_31	146	71
M2_31	135	86

Heterozygous sites called out using the diploid data also showed inconsistency for ~1% of the total SNPs (46281) in the M lines 1 (560) and 2 (37) when the lineage of every SNP is traced from M_3 till M_31 (Figure S7 and Figure S8). These are likely due to the difficulty in calling heterozygous SNPs from diploid sequencing data.

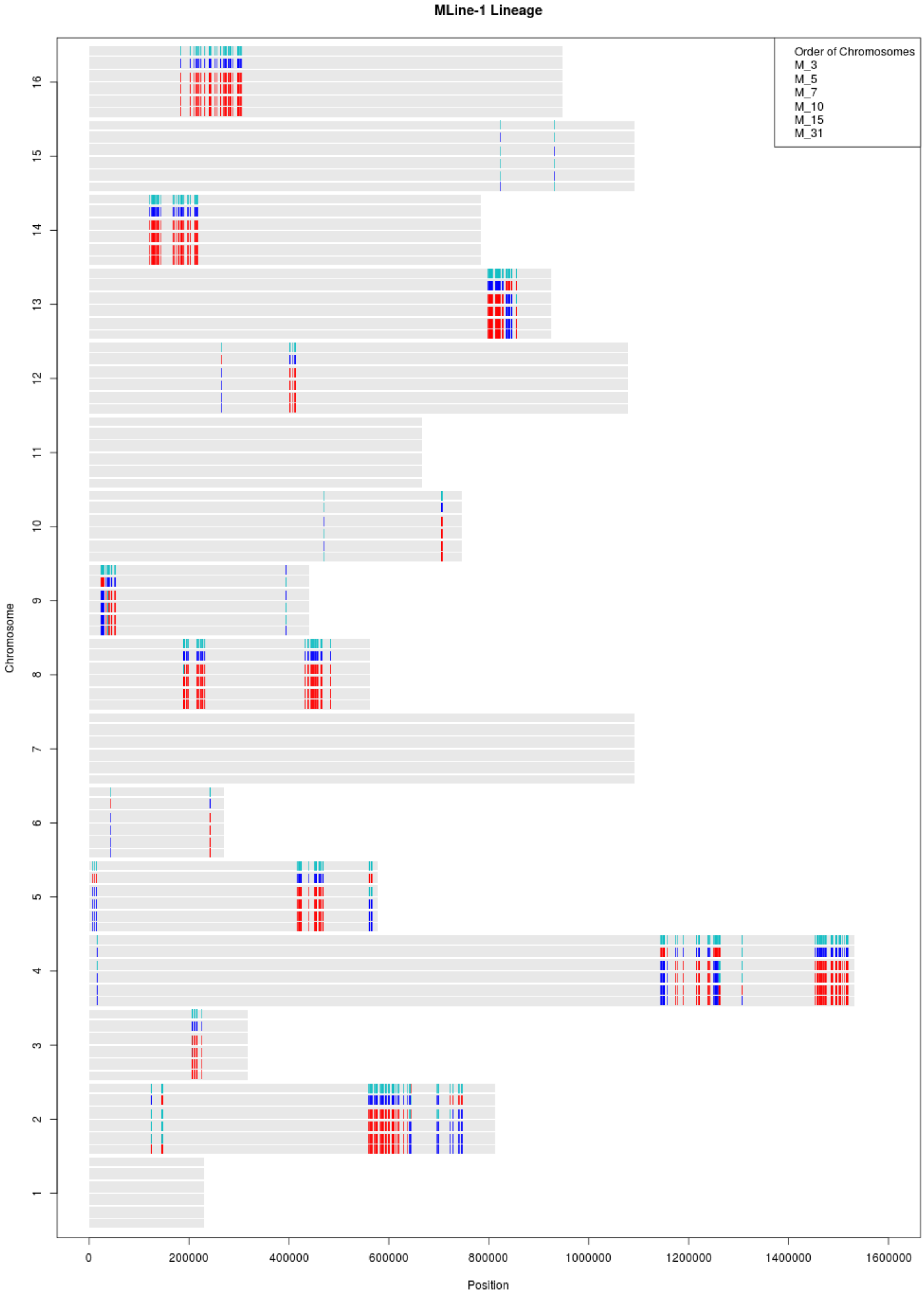

Figure S7 SNPs fixed for S288c (Red) or YJM789 (Blue) alleles that show switching to the opposite allele or towards a heterozygous state (cyan) are shown as the line 1 progresses from M_3 (top vertical bar for each chromosome) till M_31 (bottom vertical bar for each chromosome).

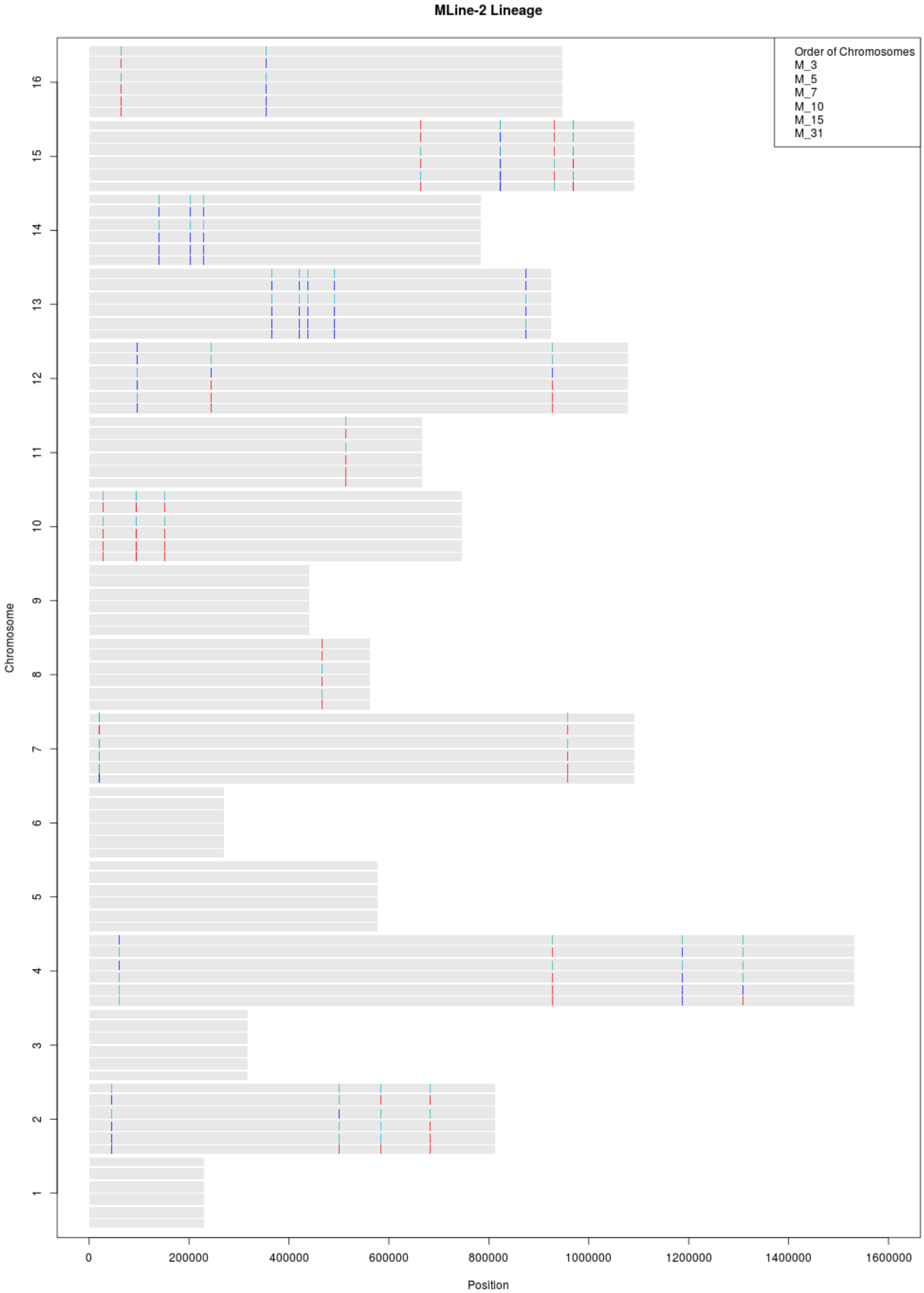

Figure S8 SNPs fixed for S288c (Red) or YJM789 (Blue) alleles that show switching to the opposite allele or towards a heterozygous state (cyan) are shown as the line 2 progresses from M_3 (top vertical bar for each chromosome) till M_31 (bottom vertical bar for each chromosome).

Literature cited

Nishant, K. T., W. Wei, E. Mancera, J. L. Argueso, A. Schlattl *et al.*, 2010 The baker's yeast diploid genome is remarkably stable in vegetative growth and meiosis. PLoS Genet 6**:** e1001109.
