# Supplementary material for: Genome Dynamics of Hybrid Saccharomyces cerevisiae During Vegetative and Meiotic Divisions
Source: G3 (Bethesda). 2017 Sep 15;7(11):3669–79. doi: 10.1534/g3.117.1135 (PMC5677154; doi:10.1534/g3.117.1135)
Supplement: Supplementary file 14 [file 3669FileS2.pdf]

File S2 Mathematical analysis of loss of heterozygosity in the meiotic lines

We estimated the expected number of heterozygous markers following each round of meiosis and mating by incorporating wild type meiotic recombination rates in our model. Recombination rates between SNPs were derived from published data of the analysis of 46 tetrads for the S288c x YJM789 cross corresponding to the parent diploid in our lines (MANCERA *et al.* 2008). Starting from the un-recombined tetrad genotype (two YJM789 and two S96), for one generation of meiosis, recombination events from one random tetrad were selected. The genotypes were swapped according to the recombination events to obtain the full recombined tetrad genotype, imitating one round of meiosis. For the next round of meiosis, two random pairs of segregants with different *MAT* locus genotypes were selected and combined to make the diploid genotype. This process is repeated for 31 rounds to obtain the final genotype expected after 31 generations. This *in-silico* recombination and selection was repeated 1000 times to obtain the expected values of heterozygosity (Table S9). Incorporation of the recombination data in the model assumes that recombination profile in the M lines does not show significant changes during propagation. To test this, pooled allele frequency data obtained from sequencing of the eight diploid M_5 lines (M1_5, M2_5, M3_5, M5_5, M6_5, M7_5, M8_5, M9_5) were plotted as a function of genome wide crossover counts (Figure S9). Switching between the S288c and YJM789 alleles are expected to occur at places where the strand containing S288c alleles exchange's with the strand containing YJM789 alleles in the hybrid. Regions showing switching between the S288c and YJM789 alleles are therefore a record of the crossover and gene conversion activities in the M lines.

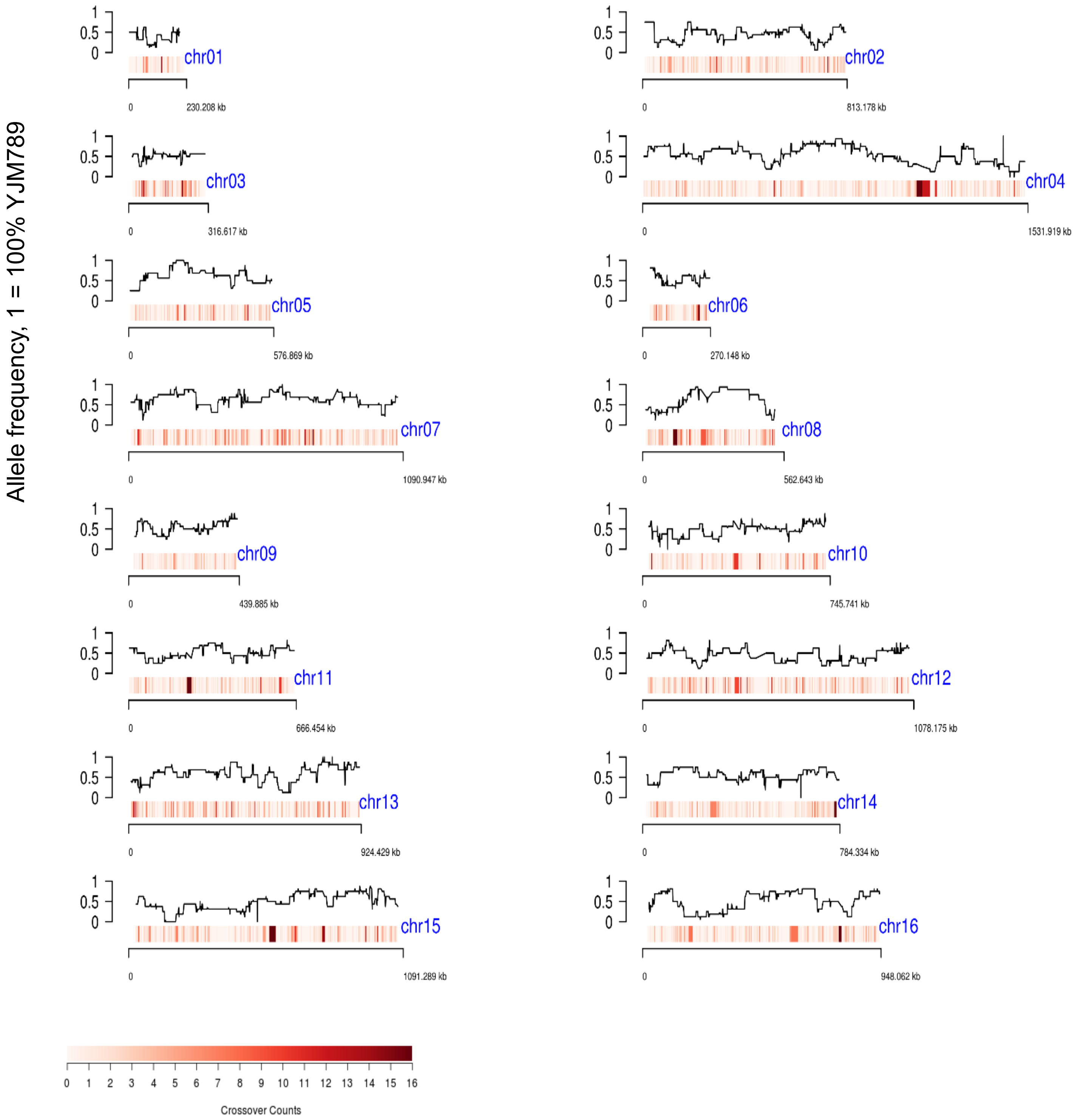

Figure S9 Allele frequency switches between S288c and YJM789 coincide with crossover peaks.

Allele frequency switches in the M_5 lines co-related well with the crossover data generated from the parent diploid strain. This result suggests crossover patterns, including hotspots and cold spots in the M lines are unlikely to have undergone major changes during the propagation of the lines for the five

meiotic generations. It is therefore reasonable to use the recombination profiles generated for the parent diploid (MANCERA *et al.* 2008) while simulating recombination at least till the M_5 lines.

Table S9 Number of heterozygous SNPs expected after each meiotic generation assuming wild type recombination

Meiotic Generation	Expected number of SNPs	Meiotic Generation	Expected number of SNPs
0	46281.00	17	186.92
1	31424.72	18	162.79
2	21471.81	19	147.09
3	14673.76	20	135.71
4	10039.27	21	128.34
5	6873.05	22	121.70
6	4749.70	23	115.74
7	3313.35	24	112.42
8	2315.94	25	109.87
9	1595.88	26	107.56
10	1139.71	27	104.51
11	788.47	28	102.85
12	575.42	29	100.99
13	435.72	30	99.48
14	340.25	31	98.24
15	265.44		
16	217.74		

Literature cited

Mancera, E., R. Bourgon, A. Brozzi, W. Huber, and L. M. Steinmetz, 2008 High-resolution mapping of meiotic crossovers and non-crossovers in yeast. Nature 454**:** 479-485.
